# ATP synthase-associated coiled-coil-helix-coiled-coil-helix (CHCH) domain-containing proteins are critical for mitochondrial function in *Toxoplasma gondii*

**DOI:** 10.1128/mbio.01769-23

**Published:** 2023-10-05

**Authors:** Madelaine M. Usey, Diego Huet

**Affiliations:** 1 Department of Cellular Biology, University of Georgia, Athens, Georgia, USA; 2 Center for Tropical and Emerging Global Diseases, University of Georgia, Athens, Georgia, USA; 3 Department of Pharmaceutical and Biomedical Sciences, University of Georgia, Athens, Georgia, USA; Albert Einstein College of Medicine, Bronx, New York, USA

**Keywords:** *Toxoplasma*, mitochondria, ATP synthase

## Abstract

Coiled-coil-helix-coiled-coil-helix (CHCH) domains consist of two pairs of cysteine residues that are oxidized to form disulfide bonds upon mitochondrial import. Proteins containing these domains play important roles in mitochondrial ultrastructure and in the biogenesis, function, and stability of electron transport chain complexes. Interestingly, recent investigations of the *Toxoplasma gondii* ATP synthase identified subunits containing CHCH domains. As CHCH domain proteins have never been found in any other ATP synthase, their role in *T. gondii* was unclear. Using conditional gene knockdown systems, we investigated two *T. gondii* ATP synthase subunits containing CHCH domains: ATPTG8 and ATPTG9. We show that these two subunits are essential for the lytic cycle as well as stability and function of the ATP synthase. Further, we illustrated that their knockdown disrupts multiple aspects of mitochondrial morphology, including ultrastructure and cristae density. Mutation of key cysteine residues in the CHCH domains also caused mis-localization of the proteins. Our work suggests that these proteins likely provide structural support to the exceptionally large *T. gondii* ATP synthase complex and that perturbations to the structural integrity of this complex result in deleterious downstream effects on the parasite mitochondrion. These investigations add to a growing body of work focused on the divergent aspects of the apicomplexan ATP synthase, which could ultimately uncover novel drug targets.

**IMPORTANCE:**

Members of the coiled-coil-helix-coiled-coil-helix (CHCH) domain protein family are transported into the mitochondrial intermembrane space, where they play important roles in the biogenesis and function of the organelle. Unexpectedly, the ATP synthase of the apicomplexan *Toxoplasma gondii* harbors CHCH domain-containing subunits of unknown function. As no other ATP synthase studied to date contains this class of proteins, characterizing their function will be of broad interest to the fields of molecular parasitology and mitochondrial evolution. Here, we demonstrate that that two *T. gondii* ATP synthase subunits containing CHCH domains are required for parasite survival and for stability and function of the ATP synthase. We also show that knockdown disrupts multiple aspects of the mitochondrial morphology of *T. gondii* and that mutation of key residues in the CHCH domains caused mis-localization of the proteins. This work provides insight into the unique features of the apicomplexan ATP synthase, which could help to develop therapeutic interventions against this parasite and other apicomplexans, such as the malaria-causing parasite *Plasmodium falciparum*.

## INTRODUCTION

The adenosine triphosphate synthase (ATP synthase) is a membrane-bound multisubunit enzyme responsible for generating cellular energy in the form of ATP. This complex consists of two distinct parts: the membrane-bound F_o_ portion and the knob-like catalytic F_1_ portion, which extends away from the membrane. The F_o_ and F_1_ portions are functionally linked via a central and peripheral stalk (1). Within eukaryotic mitochondria, the complexes of the electron transport chain (ETC) generate a proton gradient across the inner mitochondrial membrane. The gradient can be converted into rotation of the ATP synthase central stalk by proton translocation through the F_o_ portion of the enzyme. This rotation, which is counteracted by the peripheral stalk, causes conformational changes that allow for the generation of ATP from ADP and inorganic phosphate via the process of oxidative phosphorylation: a well-characterized and conserved process (2 – 4). While the general architecture and subunit composition of the ATP synthase are similarly conserved among a wide array of organisms including plants, bacteria, and mammals (5), relatively little is known about the ATP synthase in members of the apicomplexan phylum.

Apicomplexan parasites are a large group of eukaryotic pathogens that impose a significant burden on global public health. Previous work in *Toxoplasma gondii* and *Plasmodium* spp., the causative agents of toxoplasmosis and malaria, respectively, has illustrated that these parasites can shift their reliance on glycolysis or oxidative phosphorylation for ATP production to fit varying metabolic needs faced throughout their complex life cycles (6, 7). As such, studies in the mouse model of malaria, *Plasmodium berghei*, found that while disruption of the ATP synthase was only modestly detrimental to asexual blood stages, it completely blocked sexual replication during the insect stages of the life cycle (8). While *T. gondii* tachyzoites also exhibit metabolic flexibility (9 – 11), an intact ATP synthase was shown to be essential for tachyzoite survival, and it has been estimated that more than 80% of ATP in egressed tachyzoites is generated via the TCA cycle under normal conditions (6, 12).

In addition to their role in energy production, another important aspect of mitochondrial ATP synthases is their ability to form oligomeric complexes. In yeast and mammals, ATP synthase dimers have been shown to be critical for cristae formation by inducing curvature of the inner mitochondrial membrane, a process that is important for maintaining mitochondrial membrane potential and promoting efficient ATP production (13 – 15). The *T. gondii* ATP synthase was found to assemble into dimers via an extensive interface mediated primarily by apicomplexan-specific subunits (16). With a molecular weight of ~1,860 kDa (16, 17), the *T. gondii* ATP synthase dimer is significantly larger than the ~1,200 kDa dimer of yeast and mammals (18, 19), a trend which has been observed with most *T. gondii* respiratory complexes (20).

Beyond its significantly larger size, multiple studies have also found that the subunit composition of the *T. gondii* ATP synthase is highly divergent compared to other model organisms (12, 16, 21). In all, the *T. gondii* ATP synthase consists of 32 subunits, of which only 15 are canonical and have homologs in other phyla (16). The other 17 subunits are generally conserved only among mitochondriate apicomplexans. However, some are also found within other organisms of the Myzozoan clade, a phylogenetic group which includes chromerids, perkinsozoa, and apicomplexans. Further, several canonical subunits have extended apicomplexan-specific domains with functions that remain to be determined (16).

Though one of the divergent *T. gondii* ATP synthase subunits has been studied (16), the rest lack functional characterization. Interestingly, four of the novel phylum-specific F_o_ subunits have been found to contain putative coiled-coil-helix-coiled-coil-helix (CHCH) domains: *TGGT1_223040* (ATPTG6), *TGGT1_290710* (ATPTG7), *TGGT1_258060* (ATPTG8), and *TGGT1_285510* (ATPTG9). CHCH domains consist of sets of cysteine residues as CX_9_C motifs, in which the X represents any other amino acid, within separate α-helices of the protein (22). Though ATPTG7 does contain sets of cysteine residues similar to a CHCH domain motif, they are not arranged in the typical CX_9_C pattern.

In other organisms, proteins containing CHCH domains are often found as subunits of multiprotein complexes within the mitochondrion. Many proteins containing these domains are subunits of complexes I, III, and IV of the ETC where they are important for structure, assembly, optimal function, and chaperoning copper ions (22 – 24). CHCH domain proteins are also found to play roles in mitochondrial lipid transport (25), as part of the mitochondrial cristae organizing system (26), and as part of the mitochondrial ribosome (27).

The four proteins containing putative CHCH domains that were identified as subunits of the *T. gondii* ATP synthase also appear to be conserved in other apicomplexans (12, 16). Recent complexome profiling work found that ATPTG6 and ATPTG9 appear to be components of the *Plasmodium falciparum* ATP synthase (28). However, because CHCH domain proteins have never been observed as part of the ATP synthase in other organisms, it is unclear what role they play in apicomplexans. In this study, we characterized two of the *T. gondii* ATP synthase subunits containing CHCH domains: ATPTG8 and ATPTG9. Using conditional gene knockdown systems, we show that both genes are essential for the *T. gondii* lytic cycle and are critical for ATP synthase structural stability, oxidative phosphorylation, mitochondrial membrane potential maintenance, proper mitochondrial morphology, and cristae density. Further, we demonstrated that the CHCH domain cysteine residues in both ATPTG8 and ATPTG9 are essential for mitochondrial localization of both proteins. Ultimately, this investigation shows that ATPTG8 and ATPTG9 likely provide critical structural support to the massive *T. gondii* ATP synthase complex, the integrity of which is important for mitochondrial morphology and metabolism (12, 16). This study deepens our understanding of the apicomplexan ATP synthase and of the role played by the divergent subunits conserved throughout these pathogens.

## RESULTS

### *T. gondii* ATP synthase subunits containing CHCH domains are essential for the lytic cycle

**Fig 1:**
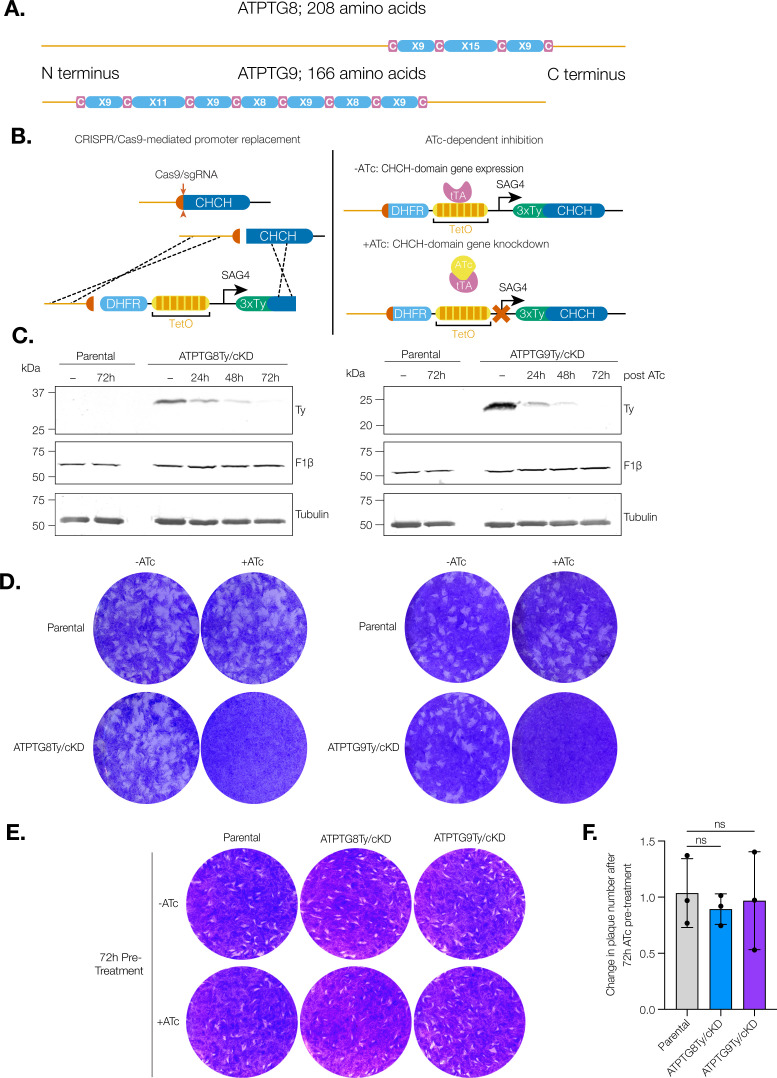
CHCH domain proteins associated with the *T. gondii* ATP synthase are essential for the lytic cycle. (A) Schematic representation of the CHCH domain size and location in ATPTG8 and ATPTG9. C represents cysteine residues and X represents any other amino acid residue. (**B)** Schematic representation of the strategy to generate ATPTG8 and ATPTG9 conditional knockdown strains (ATPTG8Ty/cKD and ATPTG9Ty/cKD). A pyrimethamine-resistant dihydrofolate reductase (DHFR) cassette, the *T. gondii* SAG4 promoter, a tetracycline-inducible operator (TetO), and an in-frame 3xTy epitope tag were inserted into the promoter region of either ATPTG8 or ATPTG9 via CRISPR/Cas9 and homology-directed repair. When anhydrotetracycline (ATc) is added to the culture medium, the tetracycline transactivator (tTA) expressed in the TATi/Δku80 parental line is no longer able to bind TetO, resulting in gene knockdown. (**C)** Lysates from parental, ATPTG8Ty/cKD, and ATPTG9Ty/cKD parasites were prepared following treatment with ATc or vehicle control (−) for the indicated amounts of time. Samples were separated via SDS-PAGE and then probed with antibodies against Ty, F1β, or tubulin. Data are representative of three biological replicates for both strains. (**D)** Plaque assay of parental, ATPTG8Ty/cKD, or ATPTG9Ty/cKD parasites grown undisturbed on a human foreskin fibroblast (HFF) monolayer in the presence of ATc or vehicle control (−) for 7–8 days. Data are representative of at least three biological replicates for both strains. (**E)** Plaque assay of parental, ATPTG8Ty/cKD, or ATPTG9Ty/cKD parasites pre-treated with ATc or vehicle control (−) for 72 h then grown undisturbed on an HFF monolayer for 7–8 days in normal culture medium. Data are representative of three biological replicates for both strains. (**F)** Quantification of plaque numbers from (**E**). Data are normalized to the vehicle control for each strain. Unpaired, two-tailed *t*-test (ns, not significant).

ATPTG8 and ATPTG9 were originally found to be associated with the *T. gondii* ATP synthase via immunoprecipitation and structural cryo-electron microscopy studies (12, 16, 21). ATPTG8 contains a single CHCH domain toward the C terminus of the gene. In contrast, ATPTG9 contains two CHCH domains (Fig. 1A), which occur in only about 8% of CHCH domain proteins (23).

To begin characterizing the roles of ATPTG8 and ATPTG9, we utilized a conditional gene knockdown system, as both genes were previously predicted to be essential for the *T. gondii* lytic cycle (29). Using CRISPR/Cas9 and homology-mediated repair, we replaced the promoter of each gene in the TATi/Δku80 strain with the SAG4 promoter, a tetracycline-inducible operator (TetO), and an in-frame N-terminal 3xTy epitope tag (Fig. 1B) (30, 31), thus creating ATPTG8Ty/cKD and ATPTG9Ty/cKD parasite lines. With this conditional gene knockdown system, when anhydrotetracycline (ATc) is added to the culture medium, the tetracycline transactivator (tTA) expressed in the TATi/Δku80 parental line is no longer able to bind TetO, inhibiting gene transcription. Once clonal populations for each line were obtained, each strain was treated with vehicle control (ethanol) or ATc for 24, 48, or 72 h. Detection of ATPTG8Ty and ATPTG9Ty by western blot shows expression of Ty-tagged proteins at the correct molecular weight in the absence of ATc and depletion of Ty signal over 72 h of ATc treatment (Fig. 1C).

We also wondered whether the promoter replacement strategy that was used to create the conditional knockdown lines altered the expression of ATPTG8 and ATPTG9 in the absence of ATc. To test this, we extracted RNA from parental, ATPTG8Ty/cKD, and ATPTG9Ty/cKD parasites and conducted quantitative reverse transcription PCR (RT-qPCR) to compare expression levels of each gene in its respective conditional knockdown strain to levels in the parental strain. We found that while promoter replacement increased the levels of both ATPTG8 and ATPTG9 transcripts, this change was only statistically significant in the ATPTG9Ty/cKD strain (Fig. S1A and B).

To confirm the essentiality of ATPTG8 and ATPTG9 for the *T. gondii* lytic cycle, we allowed ATPTG8Ty/cKD, ATPTG9Ty/cKD, and parental parasites to invade and replicate in a human foreskin fibroblast (HFF) monolayer undisturbed for 7–8 days in the presence of ATc or vehicle control. While the ability of the parental line to form plaques was unaffected by the addition of ATc, neither ATPTG8Ty/cKD nor ATPTG9Ty/cKD parasites were able to form any plaques in the presence of ATc (Fig. 1D), affirming that they are essential for the lytic cycle. However, when ATPTG8Ty/cKD and ATPTG9Ty/cKD parasites were pre-treated with ATc for 72 h before addition to the HFF monolayer with normal media, they were able to recover and form plaques (Fig. 1E). Comparison of the plaque numbers when parasites were pre-treated with vehicle vs ATc yielded no significant difference between either ATPTG8Ty/cKD or ATPTG9Ty/cKD compared to the parental strain (Fig. 1F), illustrating that viability was not significantly affected after 72 h of ATc treatment if ATc is removed from the culture medium. This experiment demonstrates that the phenotypic abnormalities discussed below are not the result of parasite death at this time point but instead reflect specific defects resulting from the loss of ATPTG8 or ATPTG9 subunits.

### ATP synthase subunits containing CHCH domains are required for structural stability and function of the complex

While ATPTG8 and ATPTG9 have previously been determined to be subunits of the *T. gondii* ATP synthase (12, 16, 17, 21), we wanted to confirm their association with the complex. To do so, we conducted anti-Ty immunoprecipitation with parental, ATPTG8Ty/cKD, and ATPTG9Ty/cKD parasite strains. The immunoprecipitation fractions were run on western blots, and membranes were probed with an antibody against the β subunit of the ATP synthase, F1β. While F1β was present in elution fractions from ATPTG8Ty/cKD and ATPTG9Ty/cKD parasites, it was not found in the elution from parental parasites (Fig. S2A and B). These data confirm studies from other groups that identified ATPTG8 and ATPTG9 as subunits of the *T. gondii* ATP synthase.

**Fig 2:**
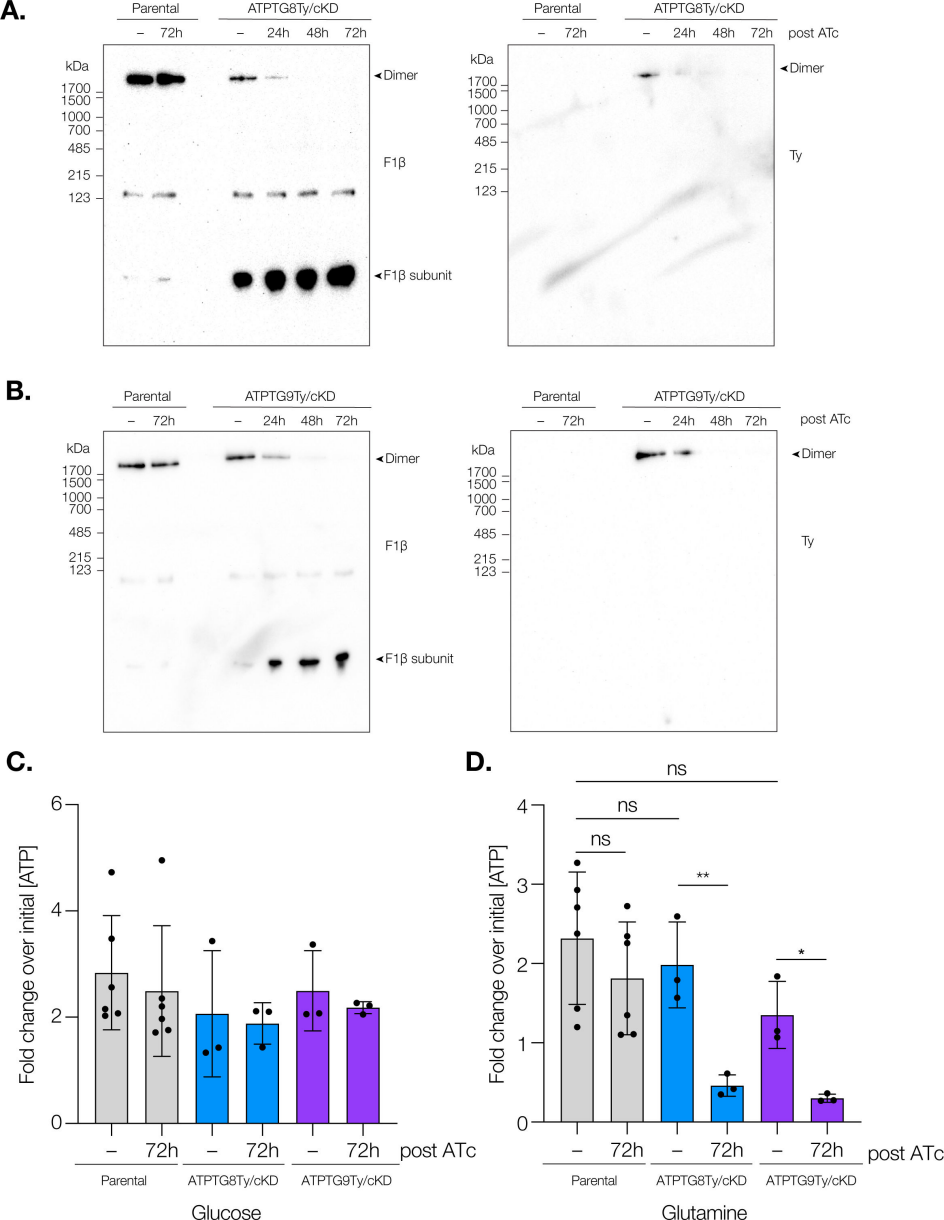
ATP synthase subunits containing CHCH domains are required for ATP synthase stability and function. (A) Lysates from parental and ATPTG8Ty/cKD parasites were prepared following treatment with ATc or vehicle control (−) for the indicated time points, then resolved by blue native PAGE (BN-PAGE), and probed for Ty and F1β. Data are representative of at least three biological representatives for both strains. (**B)** Lysates from parental and ATPTG9Ty/cKD parasites were prepared following treatment with ATc or vehicle control (−) for the indicated time points, then resolved by BN-PAGE, and probed for Ty and F1β. Data are representative of at least three biological representatives for both strains. (**C, D**) Relative ATP concentrations of parental, ATPTG8Ty/cKD, and ATPTG9Ty/cKD parasites were measured following a 72 h treatment with ATc or vehicle control. Following mechanical release, parasites were incubated for 1 h with 5 mM 2-deoxy-d-glucose (2-DG) to inhibit glycolysis and then combined with (**C**) 25 mM glucose or (**D**) 2 mM glutamine. To determine relative concentrations, ATP levels for each condition were normalized to the initial ATP readout of each strain. Data represent mean ± SD of six independent replicates for parental strains and three independent replicates for ATPTG8Ty/cKD and ATPTG9Ty/cKD strains. For one independent replicate of ATPTG8Ty/cKD, only two technical replicates were used for glucose and glutamine treatment. Unpaired, two-tailed *t*-test (ns, not significant; *P = 0.01–0.05; **P = 0.001–0.01).

We next wanted to determine the role of these two subunits in both the structure and function of this key enzymatic complex. To first investigate their structural roles, we utilized blue native-PAGE (BN-PAGE). Using an antibody against F1β, we were able to observe a strong band above 1,700 kDa in the parental strain grown in the presence of ATc or vehicle control, as well as in the conditional knockdown strains grown in vehicle control for 72 h (Fig. 2A and B). This molecular weight corresponds to the reported ~1,860 kDa mass of the *T. gondii* ATP synthase dimer (16, 17). A band of intermediate weight (~123 kDa) could also be observed in these samples, likely indicating either electrophoretic degradation or an ATP synthase assembly intermediate. In other systems, α and β subunits have been known to form a heterodimeric assembly intermediate around ~146 kDa (32). Similarly, the lowest molecular weight band observed (below 123 kDa) likely represents single β subunits. When the same blots were probed with an antibody against the Ty tag, a band above 1,700 kDa can be observed for both ATPTG8Ty/cKD and ATPTG9Ty/cKD parasites when they are treated with vehicle control (−) (Fig. 2A and B). This band corresponds to the molecular weight of the dimeric ATP synthase and further confirms that both ATPTG8 and ATPTG9 are present within this complex.

When ATPTG8Ty/cKD and ATPTG9Ty/cKD parasites were treated with ATc for increasing amounts of time, the dimeric form of the ATP synthase was depleted as observed by both F1β and Ty staining (Fig. 2A and B). Interestingly, slightly more free β subunit was consistently observed in ATPTG8Ty/cKD parasites not treated with ATc, suggesting that either modified expression of the ATPTG8 gene due to promoter replacement or the 3xTy tag at the N terminus of the protein could have caused a slight change in ATP synthase dimerization (Fig. 2A). Overall, these experiments illustrate that ATPTG8 and ATPTG9 are critical for ATP synthase structural stability in *T. gondii*.

Since ATP synthase structure was severely disrupted following CHCH domain gene knockdown, we next wanted to see whether this was associated with a concurrent disruption in ATP synthase activity. To study the metabolic effects of CHCH domain gene knockdown, we utilized a previously published metabolic assay (12). Briefly, intracellular ATPTG8Ty/cKD, ATPTG9Ty/cKD, and parental parasites were treated with ATc or vehicle for 72 h before the addition of the glycolysis inhibitor 2-deoxy-d-glucose (2-DG). In combination with the 2-DG treatment, either sufficient glucose to overcome glycolytic inhibition or sufficient glutamine to promote oxidative phosphorylation was added to the parasites. ATP levels were measured and relative ATP concentrations were determined for each treatment condition through comparison to initial ATP levels. Though ATP produced from glucose was not significantly different between strains regardless of ATc treatment (Fig. 2C), there were significant decreases in the ATP produced from glutamine when ATPTG8Ty/cKD and ATPTG9Ty/cKD parasites were treated with ATc for 72 h (Fig. 2D). Glucose contributes to ATP production by both glycolysis and oxidative phosphorylation while glutamine is only capable of contributing to oxidative phosphorylation. Thus, knockdown of these ATP synthase subunits containing CHCH domains significantly decreases the ability of *T. gondii* to generate ATP via oxidative phosphorylation.

As ATP synthase function is closely tied to mitochondrial membrane potential, we also utilized MitoTracker staining and flow cytometry to investigate how ATPTG8 or ATPTG9 knockdown affects mitochondrial membrane potential (Fig. S3). With this method, we observed that ATPTG9 knockdown over 72 h of ATc treatment resulted in a significant membrane potential depolarization compared to the parental strain. Though ATPTG8 knockdown showed decreased membrane potential compared to parental parasites, this difference was not statistically significant (*P* = 0.068) (Fig. S3). Overall, these results suggest that ATPTG8 and ATPTG9 are important for proper maintenance of the mitochondrial membrane potential.

### CHCH domain proteins are important for *T. gondii* mitochondrial morphology and volume

**Fig 3:**
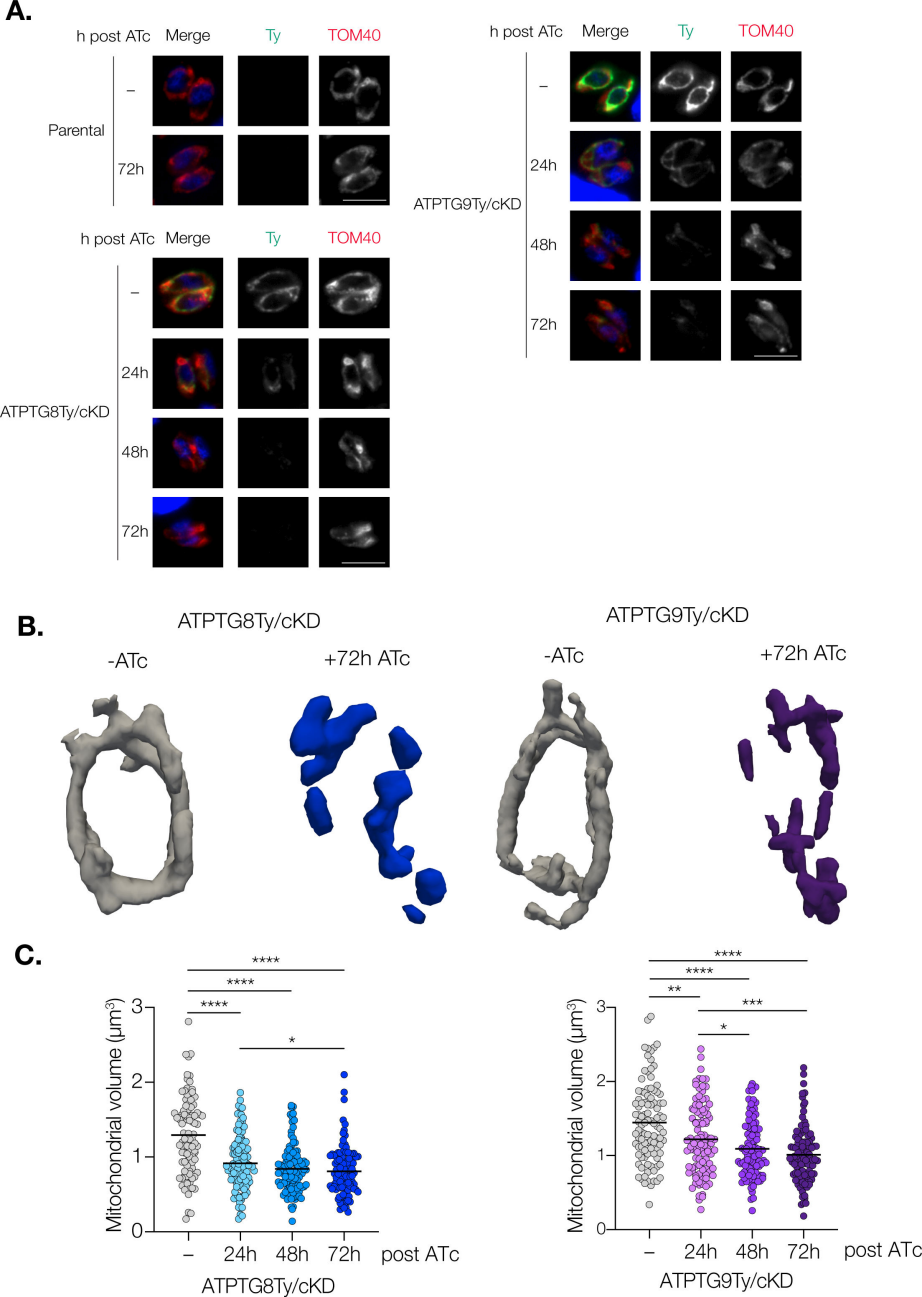
Mitochondrial morphology and volume are disrupted upon CHCH domain protein knockdown. (A) Intracellular parental, ATPTG8Ty/cKD, or ATPTG9Ty/cKD parasites were fixed and stained for DAPI (blue), Ty (green), and the mitochondrial marker TOM40 (red) following the addition of ATc or vehicle control (−) for the indicated time points. Representative images from three independent replicates. Scale bar: 5 µm. (**B, C**) Mitochondria from intracellular ATPTG8Ty/cKD and ATPTG9Ty/cKD parasites expressing SOD2-GFP and IMC1-TdTomato were analyzed via MitoGraph.** (B)** Representative 3D MitoGraph projections of mitochondria from ATPTG8Ty/cKD and ATPTG9Ty/cKD parasites following treatment with ATc or vehicle control (−) for 72 h. (**C)** Quantification of mitochondrial volumes from ATPTG8Ty/cKD and ATPTG9Ty/cKD parasites after treatment with ATc or vehicle control (−) for the indicated times. At least 100 vacuoles were measured for each condition over three independent replicates for ATPTG8Ty/cKD or two independent replicates for ATPTG9Ty/cKD. Unpaired, two-tailed *t*-test (****P < 0.0001, ***P = 0.0001–0.001, **P = 0.001–0.01, *P = 0.01–0.05).

In *T. gondii*, as well as in humans, defects in ATP synthase structure can lead to distorted, swollen, and abnormally shaped mitochondria (12, 33, 34). To investigate whether similar defects are observed upon knockdown of ATPTG8 and ATPTG9, we utilized immunofluorescence microscopy. Mitochondrial morphology was observed via staining against the outer mitochondrial membrane protein TOM40 (35). In intracellular ATPTG8Ty/cKD and ATPTG9Ty/cKD parasites treated with vehicle control, as well as in parental parasites regardless of ATc treatment, TOM40 staining consistently showed a lasso-shaped signal (Fig. 3A). This is in line with previous observations illustrating that most intracellular *T. gondii* parasites exhibit lasso-shaped mitochondria (36). However, as ATPTG8Ty/cKD and ATPTG9Ty/cKD parasites are treated with ATc for increasing amounts of time, mitochondria appear increasingly fragmented and abnormally shaped as indicated by the TOM40 staining (Fig. 3A). These data indicate that loss of ATPTG8 and ATPTG9 critically affects mitochondrial morphology.

Though immunofluorescence can be useful for investigating gross mitochondrial defects, we wanted to delve deeper into the mitochondrial abnormalities we observed upon ATP synthase-associated CHCH domain protein knockdown. To do so, we utilized mitochondrial volume analysis, a technique that has previously been used in both yeast and *T. gondii* to monitor mitochondrial morphology in three dimensions and quantify changes in mitochondrial volume (12, 37). In order to create strains amenable to mitochondrial volume analysis, we transfected both ATPTG8Ty/cKD and ATPTG9Ty/cKD parasite strains with plasmids encoding GFP fused to the mitochondrial targeting signal of SOD2 and TdTomato fused to the inner membrane complex protein IMC1 (38, 39). As *T. gondii* mitochondrial morphology changes dramatically during endodyogeny (40), the IMC1-TdTomato signal allowed us to exclude any parasites undergoing division from our analysis. Using z-stacking and the MitoGraph software (37), we were able to quantify mitochondrial volume and observe 3D changes in mitochondrial ultrastructure from SOD2-GFP signal as ATPTG8Ty/cKD and ATPTG9Ty/cKD parasites were treated with ATc over 72 h. With this approach, we also observed mitochondrial fragmentation in parasites treated with ATc from both conditional knockdown strains (Fig. 3B). Additionally, quantification of mitochondrial volumes from 100 vacuoles per timepoint illustrated that after just 24 h of ATc treatment, mitochondrial volume decreased significantly in both strains and continued to decline at 48 and 72 h timepoints as well (Fig. 3C). These results, in combination with our observations via immunofluorescence, confirm that mitochondrial fragmentation and volume loss occur upon ATPTG8 and ATPTG9 knockdown.

### Knockdown of ATP synthase-associated CHCH domain proteins results in decreased cristae density

**Fig 4:**
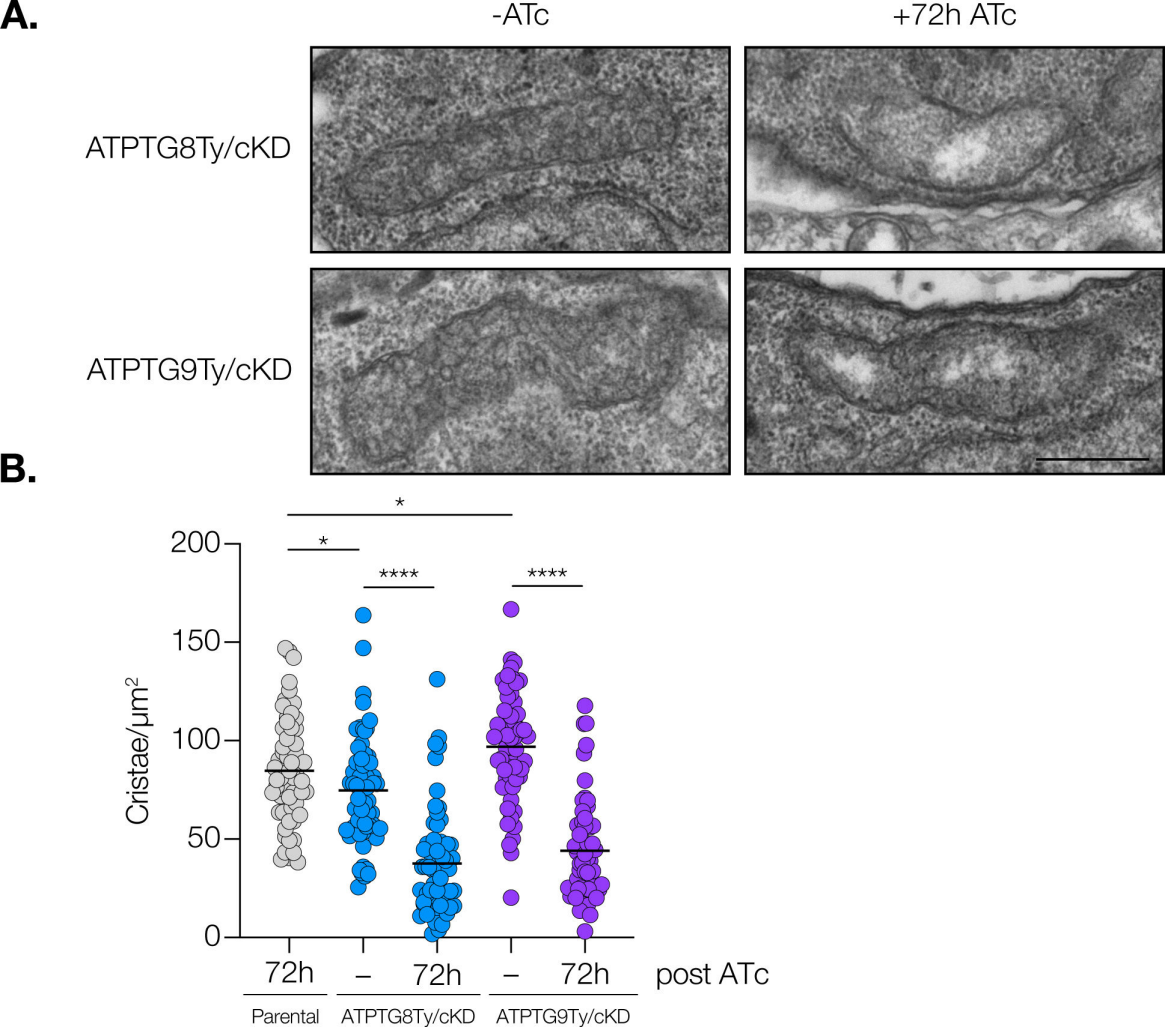
Mitochondrial cristae density decreases significantly upon CHCH domain protein knockdown. Mitochondrial cristae density of parental, ATPTG8Ty/cKD, and ATPTG9Ty/cKD parasites treated with ATc or vehicle control (−) was determined using transmission electron microscopy. (**A)** Representative electron micrographs of mitochondrial sections from ATPTG8Ty/cKD and ATPTG9Ty/cKD parasites treated with ATc or vehicle control (−) for 72 h. Scale bar: 500 nm. (**B).** Quantification of cristae/µm^2^ of mitochondrial area of parental parasites + 72 h ATc and ATPTG8Ty/cKD or ATPTG9Ty/cKD treated with ATc or vehicle control (−) for 72 h. Data represent mean ± SD for 60 sections of each condition, which were blinded prior to analysis. Unpaired, two-tailed *t*-test (****P < 0.0001, *P = 0.01–0.05).

To continue uncovering the mitochondrial ultrastructure phenotypes observed upon CHCH domain protein knockdown, we next wanted to investigate whether knockdown led to changes in mitochondrial cristae. Cristae are invaginations of the inner mitochondrial membrane associated with respiration efficiency and mitochondrial health (41). In both yeast and mammals, the ATP synthase dimer has been shown to be crucial in shaping and maintaining mitochondrial cristae (13, 42). A similar phenomenon has previously been observed in *T. gondii*, in which loss of ATP synthase integrity or its ability to assemble into higher-order structures have both disrupted proper cristae formation (12, 16). We utilized transmission electron microscopy to observe changes in cristae density between the parental strain treated with ATc and each conditional knockdown strain treated with ATc or vehicle control for 72 h. While there were some variations between the parental, ATPTG8Ty/cKD, and ATPTG9Ty/cKD strains upon treatment with vehicle control, there was a significant decrease in cristae density when ATc was added to ATPTG8Ty/cKD or ATPTG9Ty/cKD parasites for 72 h (Fig. 4A and B). Similar mitochondrial areas were investigated for all samples, except for ATPTG9Ty/cKD (Fig. S4). Overall, the depletion of ATPTG8 and ATPTG9 causes mitochondrial fragmentation, loss of mitochondrial volume, and decreased cristae density.

### CX_9_C cysteine residues are required for mitochondrial localization of ATP synthase-associated CHCH domain proteins

**Fig 5:**
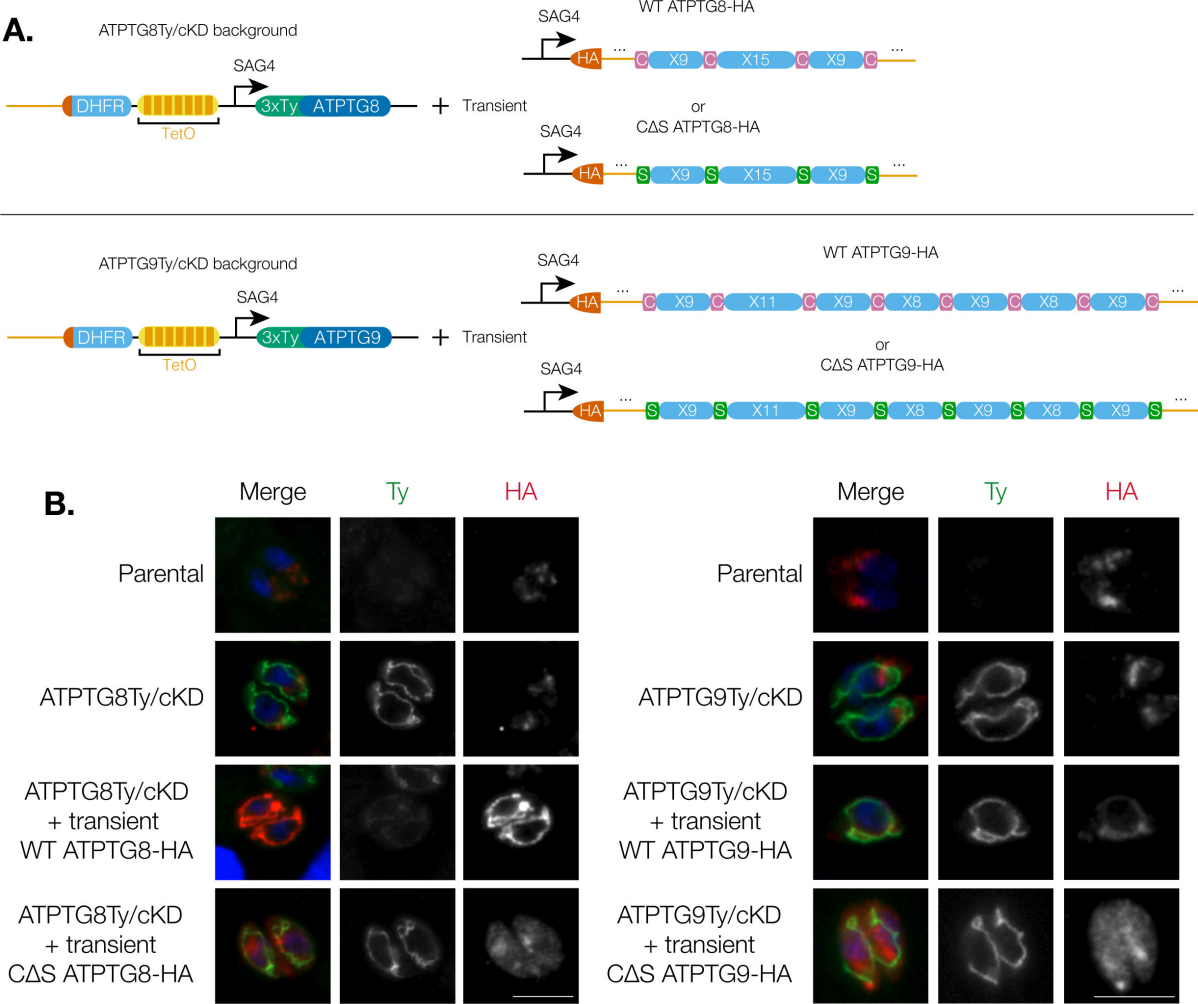
CX_9_C cysteine residues are necessary for mitochondrial localization of ATP synthase-associated CHCH domain proteins. (A) Schematic of the strategy to transiently express exogenous HA-tagged wild-type (WT) or mutant CHCH cysteineΔserine (CΔS) copies of ATPTG8 or ATPTG9 in the respective tagged conditional knockdown strain. (**B)** Immunofluorescence assays of parasites from TATi/Δku80 parental, ATPTG8Ty/cKD, or ATPTG9Ty/cKD strains transiently expressing the HA-tagged WT or CΔS copy of the respective gene, as well as un-transfected control parasites. Intracellular parasites were fixed and stained for DAPI (blue), Ty (green), and HA (red). Scale bar: 5 µm. Images are representative of three independent replicates.

A defining characteristic of CHCH domain proteins is their cysteine residues: two pairs of cysteine residues each separated by nine other amino acids in separate α-helices (22). Upon import into the mitochondrial intermembrane space (IMS), the CHCH domain cysteines are oxidized to form disulfide bonds. These intramolecular bonds have been shown to be critical for the proper folding, stability, and import of the proteins into the IMS (43 – 45). To investigate whether the cysteine residues in ATPTG8 and ATPTG9 were similarly important, we created plasmids containing either an HA-tagged wild-type (WT) coding sequence of each gene or an HA-tagged copy in which cysteine residues of the CHCH domain were mutated to serines. These plasmids were then transfected into the respective ATPTG8Ty/cKD or ATPTG9Ty/cKD strain (Fig. 5A). Within 24 h of transfection, immunofluorescence assays were utilized to observe the localization of the transiently expressed HA-tagged wildtype and cysteine∆serine CHCH domain proteins (Fig. 5B). In every parasite observed, the transiently expressed HA-tagged wild-type copy of each gene clearly localized to parasite mitochondrion, while the mutant proteins mis-localized and were instead found in the cytoplasm (Fig. 5B).

Intriguingly, when we repeatedly attempted to integrate an exogenous copy of N-terminally HA-tagged wildtype or cysteine∆serine ATPTG8 into the parasite uracil phosphoribosyltransferase (UPRT) locus (Fig. S5A), we were unable to determine the localization of these proteins by immunofluorescence assay. Stable integration into the correct locus was confirmed via PCR and sequencing (Fig. S5B and C), and the presence of HA-tagged transcripts from both the wildtype and cysteine∆serine versions of the exogenous ATPTG8 was confirmed via RT-qPCR at levels that did not differ significantly from the levels of Ty-tagged ATPTG8 in the ATPTG8Ty/cKD parental line (Fig. S5D). While HA-tagged wildtype ATPTG8 protein was observed at the correct size via western blot, no such signal could be observed for the strain expressing the cysteine∆serine version (Fig. S5E). The level of HA signal did not significantly change upon the addition of ATc for 48 h to knock down the Ty-tagged endogenous ATPTG8 gene (Fig. S5F). However, when we subjected the parasites stably expressing exogenous HA-tagged wildtype or cysteine∆serine ATPTG8 to immunofluorescence microscopy and stained for both Ty and HA, we were unable to see any HA signal above background levels (Fig. S5G). Furthermore, neither the exogenously expressed wildtype nor the cysteine∆serine ATPTG8 copies were able to complement and rescue downregulation of the endogenously Ty-tagged copy upon addition of ATc, as observed via plaque assay (Fig. S5H). Nevertheless, our results show that the cysteine residues in the CHCH domains of both ATPTG8 and ATPTG9 are critical for mitochondrial localization of both proteins.

## DISCUSSION

While the mitochondrion is commonly known as “the powerhouse of the cell,” the source of its power stems from the ATP synthase: the multisubunit enzymatic complex that generates large amounts of cellular energy in the form of ATP. The subunit composition of the ATP synthase is remarkably well-conserved among a wide range of phyla, including yeast and mammals (46). While the *T. gondii* ATP synthase contains most of the canonical subunits, it also contains 17 other largely uncharacterized, apicomplexan-specific subunits. In the present study, we characterized two novel subunits of the *T. gondii* ATP synthase that contain CHCH domains: ATPTG8 and ATPTG9.

Using conditional gene knockdown systems, we validated that both genes are essential for the *T. gondii* lytic cycle, as was predicted by a genome-wide CRISPR screen (29). Furthermore, we were able to determine that both genes are critical for ATP synthase structural stability as well as function. Though oxidative phosphorylation was significantly decreased, knockdown of either CHCH domain protein had no effect on the ability of the parasites to generate ATP from glucose, illustrating their metabolic flexibility. These observations of ATP synthase destabilization and decreased oxidative phosphorylation fit with findings in yeast, in which many CHCH domain proteins are found to play a role in the structural integrity, assembly, and function of respiratory chain complexes (23, 24). It has been hypothesized that most CHCH domain proteins act as structural scaffolds for large mitochondrial complexes. This may be due to the CHCH domain motif serving as a stable, yet easily modifiable, building block that can be folded through a conserved mechanism and regulated by mitochondrial REDOX state (23). While CHCH domain proteins have never previously been observed as subunits of the ATP synthase in any other organism, ATPTG8 and ATPTG9 likely provide additional structural support to the exceptionally large *T. gondii* ATP synthase complex that contains nearly double the number of subunits found in yeast and mammalian complexes (18, 19). A potential role of these proteins in other mitochondrial complexes or in the assembly of the ATP synthase is unlikely, as both were only found to exist as part of an ~1,800 kDa complex by BN-PAGE.

Although *T. gondii* tachyzoites can continue to generate ATP via glycolysis when ATP synthase function is disrupted, we also demonstrated that ATP synthase structural stability is important for maintaining proper mitochondrial membrane potential. Previous studies in mouse neurons illustrated that when OSCP, an ATP synthase subunit involved in stabilization of the complex, was downregulated, the cells experienced membrane potential depolarization (47). The destabilization of the complex eventually leads to the dissociation of the F1 portion and the formation of a channel within the c ring of the ATP synthase, which allows ions to leak through (47). It is possible that downregulation of ATPTG8 and ATPTG9 could destabilize the *T. gondii* ATP synthase in a way that allows ion leak across the inner mitochondrial membrane, thus resulting in the observed membrane potential depolarization. An alternative explanation is that a key function of the *T. gondii* ATP synthase is the maintenance of the mitochondrial membrane potential. In eukaryotic mitochondria, the ATP synthase can shift its function from ATP synthesis to ATP hydrolysis based on membrane potential and ATP concentration (48). In the bloodstream form of the protozoan parasite *Trypanosoma brucei*, which does not generate energy via oxidative phosphorylation, the ATP synthase is essential to parasite survival as it operates in a hydrolytic manner to maintain mitochondrial membrane potential (49, 50). Although we know *T. gondii* utilizes oxidative phosphorylation for ATP production (6), perhaps a balance of ATP synthase complexes functioning in both synthetic and hydrolytic directions is necessary for mitochondrial health and parasite survival.

Additionally, we found that knockdown of both genes caused mitochondrial fragmentation and a sharp decrease in mitochondrial volume. Similar mitochondrial defects in yeast have been observed when three different CHCH domain proteins were knocked out, leading to swollen, irregular, shorter, or less interconnected mitochondrial tubules (24). Furthermore, we showed that knockdown of both ATPTG8 and ATPTG9 resulted in significant reduction of mitochondrial cristae density. The bulbous mitochondrial cristae in *T. gondii* are shaped by pentagonal pyramid ATP synthase complexes (16) in contrast to the mammalian lamellar cristae that are shaped by a large ATP synthase dimer angle (13). Our work adds to a growing volume of evidence illustrating that disrupting both the ATP synthase dimer and pentagonal pyramids results in a loss of cristae integrity in *T. gondii* (12, 16).

Though our study illustrates that CHCH domain proteins play important roles in the *T. gondii* mitochondrion, the mechanism by which these proteins are imported and stabilized within the parasite’s mitochondrion remains unclear. In yeast, a CHCH domain protein termed Mia40 (or CHCHD4 in humans) resides in the IMS and assists in the oxidative folding of other CHCH domain proteins following their mitochondrial import. Interactions between CHCH domain proteins and Mia40 are mediated by an intermembrane targeting sequence (ITS). The ITS consists of critical hydrophobic residues in an amphipathic helix either upstream or downstream of one of the CHCH domain cysteine residues, and the majority of CHCH domain proteins lack canonical N terminal mitochondrial matrix targeting signals (23, 51). Although ATPTG8 and ATPTG9 are not predicted to contain matrix targeting signals, they contain putative ITS motifs. Tagging either gene at the N terminus did not affect their mitochondrial localization, confirming their lack of matrix targeting signals. Ultimately, through its interaction with imported CHCH domain proteins, Mia40 is able to oxidize key cysteine residues, resulting in the formation of the two disulfide bonds (52). Oxidized Mia40 is then regenerated through interaction with the flavin-linked sulfhydryl oxidase Erv1 in yeast (or ALR in humans) (53, 54).

Intriguingly, many protozoan parasites, including apicomplexans, express CHCH domain proteins but seem to lack Mia40 homologs (55). It has been hypothesized that Erv1 may have functioned independently as part of an ancestral import pathway for CHCH domain proteins in early eukaryotic lineages (56). However, complementation studies attempting to replace yeast Mia40 with protozoan Erv1 have failed to validate this (57). While three putative Erv1 homologs have been identified in the *T. gondii* genome (35), the identity of the oxidoreductase responsible for folding CHCH domain proteins in these parasites remains unknown.

Our work demonstrates that the cysteine residues within ATPTG8 and ATPTG9 are critical for their proper mitochondrial localization; while transient expression of wild-type ATPTG8 or ATPTG9 showed consistent mitochondrial localization, transiently expressed cysteine∆serine copies of both genes appeared as a diffuse cytoplasmic signal. When we stably expressed wildtype and cysteine∆serine copies of ATPTG8 from the parasite UPRT locus, both transcripts and protein of wild-type ATPTG8 could be detected, while only transcripts of the cysteine∆serine version could be observed. These observations are supported by previous studies in which CHCH domain cysteine mutants appear to exhibit disrupted localization, co-localize with lysosomes, or be degraded via other methods (42, 43). These data emphasize the importance of the CHCH domain cysteines in the localization and stability of ATPTG8 and ATPTG9 and further suggest that *T. gondii* CHCH domain proteins must interact with a currently undetermined oxidoreductase to be properly folded and maintained within the mitochondrion. As the putative parasite oxidoreductase likely diverges significantly from its human counterpart, it could represent another novel therapeutic target and its identification warrants future study.

Just as the CHCH domain oxidoreductase folding partner in these parasites eludes identification, we also lack knowledge on how CHCH domain proteins are regulated in *T. gondii*. When we attempted to stably express HA-tagged wild-type exogenous ATPTG8 from the UPRT locus in the ATPTG8Ty/cKD parasite line, we were able to observe HA-tagged ATPTG8 transcripts and protein. However, this stably expressed protein did not seem to localize to the mitochondrion at levels detectable via immunofluorescence microscopy, nor did it complement knockdown of the endogenously Ty-tagged gene. Our inability to observe wild-type ATPTG8-HA via immunofluorescence, despite detecting the protein by western blot, could be due to decreased sensitivity of immunofluorescence assays as compared to western blotting. Low protein levels of wild-type exogenous ATPTG8-HA may have resulted in a mitochondrial signal that was indistinguishable above background immunofluorescence levels. On the other hand, some CHCH domain proteins have been shown to undergo alternative splicing, and splice-site mutations have been associated with cancers and diseases in humans by reducing functional CHCH domain protein expression (58, 59). Thus, fusing the exons together to create the exogenous HA-tagged gene construct might have caused this copy to be preferentially downregulated or inefficiently imported into the mitochondrion as compared to splice variants of the endogenous gene copy. Furthermore, translocations of CHCHD7 and mutations in untranslated regions surrounding CHCHD2 have been associated with human disease (58, 59). Perhaps, the altered genomic location of our exogenous ATPTG8 disrupted its ability to function normally, preventing it from complementing the downregulation of the endogenous copy. Overall, these observations beg further investigation into the ways that CHCH domain proteins are regulated in *T. gondii*.

Additionally, while there appears to be a great deal of conservation in terms of ATP synthase subunit composition between *T. gondii* and the related apicomplexans of the genus *Plasmodium* spp., very little is known about the ATP synthase in this group of malaria-causing parasites. As the ATP synthase has been shown to be essential for survival of the insect stages of *Plasmodium berghei* (8), drugs targeting this complex could block malaria transmission without affecting the mammalian host. However, additional characterization of this important complex in *Plasmodium* spp. is needed.

In summary, our work provides the first insight into the roles played by ATP synthase-associated CHCH domain proteins and increases the depth of knowledge surrounding the divergence of the apicomplexan ATP synthase. Our proposed role for ATPTG8 and ATPTG9 is that they function as structural scaffolds to maintain the large *T. gondii* ATP synthase complex, the structural integrity of which has been shown to be critical for oxidative phosphorylation and the maintenance of overall mitochondrial health (12, 16). Future work remains to characterize other putative CHCH domain proteins in *T. gondii* (Table 1), determine the identity of the oxidoreductase involved in their mitochondrial import, and elucidate potential regulatory mechanisms of this important class of proteins. Because novel drugs against these parasites are needed to combat constantly evolving resistance against current therapeutics, characterizing essential, divergent parasite proteins can offer new opportunities for future drug discovery.

**TABLE 1:** Putative CHCH domain proteins in *T. gondii*

Gene ID	ToxoDB annotation	CRISPR score	LOPIT	Protein size (aa)	CHCH domain	Notes
TGGT1_203460	Hypothetical protein	−3.5	Mitochondrion soluble	119	C9C17C9C (51–89)	Homology to TRIAP1/Mdm35, a protein involved in mitochondrial lipid homeostasis (BLASTp E: 2e−05)
TGGT1_208300	Hypothetical protein	−1.9	Mitochondrion soluble	105	C9C10C9C (65–96)	
TGGT1_223040	Hypothetical protein	−4.5	Mitochondrion membranes	239	C9C16C9C (168–205)	Identified as subunit of ATP synthase by this study and others (12, 16, 17)
TGGT1_213940	CHCH domain-containing protein	−2.2	Mitochondrion soluble	149	C9C9C9C (111–141)	Homology to human CHCHD2/CHCHD10 (BLASTp E: 0.002)
TGGT1_240550	Putative copper chaperone COX17-1	−2.7	Mitochondrion soluble	77	C9C8C9C (39–68)	
TGGT1_254260	COX19 cytochrome *c* oxidase assembly family protein	−3.3	Mitochondrion soluble	256	C9C32C9C (29–82)	
TGGT1_258060	Putative myosin heavy chain	−4.1	Mitochondrion membranes	208	C9C15C9C (129–165)	Identified as subunit of ATP synthase by this study and others (12, 16, 17)
TGGT1_268740	Hypothetical protein	−1.6	Mitochondrion soluble	187	C9C10C9C (91–122)	
TGGT1_285510	Hypothetical protein	−1.9	Mitochondrion membranes	166	C9C11C9C8C9C8C9C (14–84)	Identified as subunit of ATP synthase by this study and others (12, 16, 17, 21)
TGGT1_286070	Hypothetical protein	−3.4	Mitochondrion soluble	86	C9C29C9C (18–68)	
TGGT1_290710	Hypothetical protein	0.7	Not localized	236	C9C9C11C** (193–225)	Identified as subunit of ATP synthase by (16, 17). **Does not contain a typical twin CX9C motif
TGGT1_297465	Hypothetical protein	0.7	Not localized	94	C9C19C9C (27–67)	
TGGT1_311390	tRNA (guanine(9)-N(1))- methyltransferase	1.4	Not localized	605	C9C9C9C (317–347)	
TGGT1_313160	Hypothetical protein	−4	Not localized	138	C9C11C9C (51–83)	
TGGT1_320140	Putative ubiquinol-cytochrome *c* reductase hinge protein	−3.7	Mitochondrion membranes	89	C9C13C9C (41–75)	Identified as CIII subunit QCR6/Hinge protein (17, 60)

List of CHCH domain proteins primarily generated via ToxoDB BLASTp analysis based on data sets of CHCH domain proteins in other organisms (22, 23). LOPIT data: (61). CRISPR scores: (29).

## MATERIALS AND METHODS

### Parasite culture

RH/TATi/Δku80 (30) tachyzoites and their derivatives were maintained in human foreskin fibroblasts (HFFs) (ATCC, cat. no. SCRC-1041). Strains were cultured at 37°C and 5% CO_2_ in DMEM supplemented with 2 mM glutamine (GeminiBio, cat. no. 400-106) and 3% heat-inactivated fetal calf serum (IFS).

### Plasmid generation

To generate the ATPTG8Ty/cKD and ATPTG9Ty/cKD strains, the pU6-Universal plasmid (Addgene, cat. no. 52694) was digested using the BsaI restriction enzyme and then sgRNAs targeting the N terminus of each gene (P1 and P2 for ATPTG8 or P5 and P6 for ATPTG9) were inserted into the plasmid using Gibson assembly. A repair template encoding a pyrimethamine-resistant copy of the dihydrofolate reductase (DHFR) cassette, the *T. gondii* SAG4 promoter, a tetracycline-inducible operator, and a 3xTy epitope tag was amplified from the DHFR-SAG4-TetO7-3xTy plasmid (a kind gift from Silvia Moreno) using P3 and P4 for ATPTG8Ty/cKD or P7 and P8 for ATPTG9Ty/cKD.

To modify the ATPTG8Ty/cKD strain so that it would transiently express either wildtype or cysteine∆serine copies of ATPTG8, a plasmid that contained the SAG4 promoter, an N-terminal HA tag, the ATPTG8 CDS, and 1,000 bp of the endogenous ATPTG8 3′ UTR (SAG4-HA-ATPTG8WT) was assembled via Gibson assembly. To create a plasmid encoding a cysteine∆serine mutant copy of the gene (SAG4-HA-ATPTG8CtoS), a portion of the ATPTG8 CDS was digested out with EcoNI and StuI. An oligonucleotide carrying the portion of the ATPTG8 CDS with all cysteine to serine mutations (P9) was amplified with P10 and P11 and inserted using Gibson assembly.

A similar approach was used for ATPTG9: a plasmid containing the SAG4 promoter, an N-terminal HA tag, the ATPTG9 CDS, and 1,000 bp of the endogenous ATPTG9 3′ UTR (SAG4-HA-ATPTG9WT) was assembled via Gibson assembly. To create a plasmid encoding a cysteine∆serine mutant copy of the gene (SAG4-HA-ATPTG9CtoS), a portion of the ATPTG9 CDS was digested out with KpnI and EcoRI. An oligonucleotide carrying the portion of the ATPTG9 CDS with all cysteine to serine mutations (P12) was amplified with P13 and P14 and inserted using Gibson assembly.

For stable integration of exogenous wildtype or cysteine∆serine copies of ATPTG8 into the ATPTG8Ty/cKD parasite strain UPRT locus (TGGT1_312480), the repair template was amplified from SAG4-HA-ATPTG8WT or SAG4-HA-ATPTG8CtoS plasmids using P15 and P16, which carry 40 bp of homology to the *T. gondii* UPRT gene. Additionally, the pU6-Universal plasmid was digested using the BsaI restriction enzyme then an sgRNA (P17 and P18) targeting the UPRT locus was inserted into the plasmid using Gibson assembly.

### Parasite strain generation

To create the ATPTG8Ty/cKD and ATPTG9Ty/cKD strains, TATi/Δku80 parasites were transfected as previously described (62). These transfections were conducted with 25–50 µg of pU6-Universal plasmid encoding Cas9 and an sgRNA targeting the N terminus of each gene. The repair oligonucleotide co-transfected with this plasmid encoded a pyrimethamine-resistant DHFR cassette, the *T. gondii* SAG4 promoter, tetracycline-inducible operator, and an in-frame 3xTy epitope tag flanked on either end by 40 bp of homology to the 5′ UTR or N terminus of the gene. Pyrimethamine (Sigma Aldrich, cat. no. 46706-250MG) was used at 3 µM to select parasites containing the integration. Expression of the Ty tag and its downregulation after addition of 0.5 µg/mL anhydrotetracycline (ATc) were confirmed via immunofluorescence assay and western blot. Positive integrants were subcloned via serial dilution to obtain a monoclonal population.

For mitochondrial volume analysis experiments, both the ATPTG8Ty/cKD and ATPTG9Ty/cKD strains were transfected with pT8mycSOD2(SPTP)GFPmycHX (39) and TubIMC1TdTomato-CAT plasmids (38). Double-positive parasites were sorted via FACS to isolate clonal populations stably expressing both fluorescent proteins.

For localization of transiently expressed wildtype or cysteine∆serine ATPTG8 or ATPTG9, ~50–100 µg of SAG4-HA-ATPTG8WT or SAG4-HA-ATPTG8CtoS plasmids were transfected into the ATPTG8Ty/cKD line, while ~50–100 µg of SAG4-HA-ATPTG9WT or SAG4-HA-ATPTG9CtoS plasmids were transfected into the ATPTG9Ty/cKD line. Immediately after transfection, 40 µL of parasites were added to coverslips pre-seeded with HFF cells for monitoring of transient HA expression within 24 h of transfection.

To create strains stably expressing either wildtype or cysteine∆serine copies of ATPTG8, the ATPTG8Ty/cKD strain was transfected with 25–50 µg of pU6-Universal plasmid encoding Cas9 and an sgRNA targeting the UPRT locus (TGGT1_312480). The plasmid was co-transfected with a repair template that had 40 bp of homology on both ends to the UPRT locus and encoded either a wildtype copy of ATPTG8 or a copy in which all cysteines of the CHCH domain were mutated to serines. This exogenous copy was under the control of the SAG4 promoter, contained an in-frame HA epitope tag appended to the N terminus, and was followed by 1,000 bp of the endogenous 3′UTR. To select for positive transfectants, the thymidylate synthase inhibitor 5-fluoro-2′-deoxyuridine (FUDR) (Sigma Aldrich, cat. no. F0503-100MG) was used at 5 µM. Integration of the repair template into the UPRT locus was confirmed via PCR with P19 and P20. HA tag expression was determined via western blot.

### Plaque assays

To determine effects of CHCH domain protein knockdown on the *T. gondii* lytic cycle, 500 TATi/Δku80 parental, ATPTG8Ty/cKD, or ATPTG9Ty/cKD parasites were added to each well of a 6-well plate pre-seeded with HFF cells and left undisturbed for 7–8 days. Wells were treated with 0.5 µg/mL ATc or ethanol (vehicle control) for the entirety of the experiment.

For the pre-treatment plaque assays, parasites were first allowed to invade a host cell monolayer for 24 h before ATc or vehicle control was added to the culture medium. At 24 h after the addition of ATc or vehicle control, when the parasites lysed, they were passed onto a fresh host cell monolayer with ATc or vehicle control present in the culture medium. After 48 h when the parasites began to lyse, they were collected and 500 parasites were added to each well of a 6-well plate in normal parasite culture medium. After 7–8 days, wells were washed with PBS, fixed in 95% ethanol for 10 min, and then stained with a crystal violet solution (2% crystal violet, 0.8% ammonium oxalate, 20% ethanol) for 5 min. Wells were subsequently washed again and then scanned for analysis. Plaques were quantified manually using Fiji.

### Western blotting

To prepare samples for western blotting, pellets containing approximately 2 × 10^7^ parasites were resuspended in 2X Laemmli buffer (20% glycerol, 5% 2-mercaptoethanol, 4% SDS, 0.02% bromophenol blue, 120 mM Tris-HCl pH 6.8) and then boiled at 100°C for 5 min. Following separation by SDS-PAGE, proteins were transferred to nitrocellulose membranes and probed with mouse anti-Ty (62), rabbit anti-HA (Cell Signaling Technologies, cat. no. 3724S), mouse anti-tubulin (Developmental Studies Hybridoma Bank at the University of Iowa, cat. no. 12G10), rabbit anti-IMC6 (a kind gift from Peter Bradley), or rabbit anti-F1β (Agrisera, cat. no. AS05 085). For signal detection using an Odyssey infrared imager (LI-COR Biosciences), donkey anti-mouse IgG conjugated to IRDye 800CW (VWR, cat. no. 102673-332) or goat anti-rabbit IgG conjugated to IRDye 680RD (VWR, cat. no. 102673-410) were used as secondary antibodies. For detection via X-ray film, membranes were incubated with either goat anti-mouse IgG conjugated to HRP (VWR, cat. no. 102646-160) or goat anti-rabbit IgG conjugated to HRP (VWR, cat. no. 102645-182) secondary antibodies. After incubation with enhanced chemiluminescence (ECL) substrate (VWR, cat. no. PI32209), autoradiography film (MTC Bio, cat. no. A8815) was exposed to the membrane and developed.

### Quantitative reverse transcription PCR

RNA was extracted from lysed parasites using the Zymo Quick-RNA MiniPrep kit (VWR, cat. no. 76020-636). RT-qPCR was conducted using primers P21-28 (Table S1) and the Luna Universal One-Step RT-qPCR kit (VWR, cat. no. 103307) in an iCycler thermal cycler (Bio-Rad). Relative quantification analysis was conducted using the 2^−∆∆*Ct*^ method.

### Anti-Ty immunoprecipitation

Before beginning immunoprecipitation, 60 µg of anti-Ty antibody (62) was coupled to 1 mg of Pierce Protein G Magnetic Beads (Thermo Scientific, cat. no. 88848). Parasites from parental, ATPTG8Ty/cKD, or ATPTG9Ty/cKD lines were lysed at 4° for 5 min in a buffer containing 150 mM NaCl, 20 mM Tris pH 7.6, 1% Triton X-100, 0.1% SDS and supplemented with 1× HALT Protease and Phosphatase Inhibitor (VWR, cat. no. PI78440). Lysates were clarified via centrifugation at 21,000 × *g* for 5 min at 4°C. The supernatant was incubated with the previously prepared anti-Ty-coupled Protein G beads for 1 h at 4°C. To elute bound proteins, beads were incubated with 150 ng/µL of Ty peptide (Genescript) suspended in lysis buffer. Immunoprecipitation fractions were then resolved by western blotting.

### Blue native polyacrylamide gel electrophoresis

For BN-PAGE, 2 × 10^7^ parasites were solubilized with 2.5% digitonin in a solution containing 1× NativePAGE sample buffer (Thermo Fisher Scientific, cat. no. BN2008). To accurately estimate the molecular weight of large membrane-bound complexes, 50 µg of bovine heart mitochondria (Abcam, cat. no. ab110338) was solubilized under the same conditions as the parasite samples for use as a standard (28). Following separation on a NativePAGE 3%–12% Bis Tris protein gel (Thermo Fisher Scientific, cat. no. BN1001BOX), proteins were transferred to a PVDF membrane and first incubated with mouse anti-Ty primary antibody (62) and then a goat anti-mouse IgG secondary antibody conjugated to HRP (VWR, cat. no. 102646-160). Following incubation with enhanced chemiluminescence (ECL) substrate (VWR, cat. no. PI32209), membranes were developed using a BioRad ChemiDoc Imaging System. After exposure of the Ty signal, membranes were stripped according to manufacturer instructions using Restore Western Blot Stripping Buffer (VWR, cat. no. PI21059) and then incubated with rabbit anti-F1β (Agrisera, cat. no. AS05 085) followed by a goat anti-rabbit IgG secondary antibody conjugated to HRP (VWR, cat. no. 102645-182). F1β signal was then captured using the same methods as Ty.

### Cellular ATP concentration measurements

To measure the ATP concentration of the parasites, HFFs were infected with parental, ATPTG8Ty/cKD, or ATPTG9Ty/cKD strains and treated with vehicle control (ethanol) or 0.5 µg/mL ATc for 72 h. As previously described (12), the monolayer was first washed with PBS to remove any extracellular parasites. Fluorobrite DMEM (Thermo Fisher Scientific, cat. no. A1896701) containing 1% IFS and HALT protease inhibitors (VWR, cat. no. PI78440) was added to the cells containing intracellular parasites before they were scraped and syringe-released. Following removal of host cell debris, parasites were pelleted, washed in DMEM free from glucose and glutamine (Fisher Scientific, cat. no. A1443001), and then resuspended in DMEM free from glucose and glutamine to a final concentration of 6 × 10^6^ parasites/mL. To determine the initial ATP concentration for each strain, 50 µL of each parasite solution was added to the wells of a 96-well PCR plate with 50 µL of DMEM free from glucose and glutamine before immediately being flash frozen in liquid nitrogen. Additionally, 50 µL of each parasite solution was added to a plate along with equal amounts of the following compounds at the listed final concentrations: 5 mM 2-deoxyglucose (Sigma Aldrich, cat. no. D6134-5G) + 25 mM glucose (Sigma Aldrich, cat. no. G7021-100G) or 5 mM 2-deoxyglucose + 2 mM glutamine (Sigma Aldrich, cat. no. G8540-100G). Parasites were allowed to incubate with each solution for 1 h at 37°C, 5% CO_2_ before being flash frozen. To determine the ATP concentration in each condition, 100 µL of CellTiter-Glo reagent (Promega, cat. no. G7572) was added to the wells while samples thawed at room temperature for 1 h. After thawing, 100 µL of each sample was added to a white, flat-bottom 96-well plate and then measured using a Molecular Devices SpectraMax i3x microplate reader. All samples and conditions were conducted in triplicate, and ATP levels for each strain were normalized to the initial concentration. Six biological replicates were obtained from the parental strain, and three replicates were obtained for ATPTG8Ty/cKD and ATPTG9Ty/cKD strains.

### Immunofluorescence assays

For the immunofluorescence assays, glass coverslips pre-seeded with HFFs were infected with approximately 40 µL of freshly lysed parasites. While the parasites were intracellular, cells were fixed in 4% paraformaldehyde for 15 min at 4°C. Following fixation, cells were permeabilized for 8 min using a solution of 0.25% Triton X-100 and then blocked for 10 min in a solution of PBS with 5% IFS and 5% normal goat serum. Cells were then stained with mouse anti-Ty (63), rabbit anti-Tom40 (a kind gift from Giel van Dooren), or rabbit anti-HA (Abcam, cat. no. ab9110) primary antibodies for 1 h. Subsequently, cells were stained with Alexa-488-conjugated goat-anti-mouse (Invitrogen, cat. no. A32723) and Alexa-647-conjugated goat-anti-rabbit (Invitrogen, cat. no. A32733), Alexa-488-conjugated goat-anti-rabbit (Invitrogen, cat. no. A32731), or Alexa-594-conjugated goat-anti-mouse (Invitrogen, cat. no. A11005) secondary antibodies. Hoechst (Santa Cruz Biotechnology, cat. no. sc-394039) was used to stain cell nuclei. Coverslips were mounted onto slides with Prolong Diamond (Thermo Fisher, cat. no. P36961). Images were acquired using an ECHO Revolve microscope and the ECHO Pro application. Image analysis and processing were conducted using Fiji, Adobe Photoshop 2022, and Adobe Illustrator 2022.

### Flow cytometry

To quantify membrane potential changes when ATPTG8 or ATPTG9 genes were knocked down, intracellular tachyzoites from parental, ATPTG8Ty/cKD, or ATPTG9Ty/cKD strains were treated with ATc or vehicle control (ethanol) for 72 h and then stained with 50 nM MitoTracker DeepRed (Life Technologies, cat. no. M22426) for 1 h at 37°C and 5% CO_2_. For FCCP and vehicle control (DMSO) samples, intracellular parental tachyzoites were stained with a solution containing 50 nM MitoTracker DeepRed (Life Technologies, cat. no. M22426) and 10 µM carbonyl cyanide 4-(trifluoromethoxy)phenylhydrazone (FCCP) (Sigma Aldrich, cat. no. C2920-10MG) or an equivalent amount of DMSO (vehicle control). After 1 h, parasites were syringe-released, filtered, and pelleted before washing once with PBS (10 µM FCCP or DMSO were included in PBS for the FCCP and DMSO samples). Samples were resuspended in PBS or PBS containing 10 µM FCCP or DMSO for analysis via flow cytometry. Membrane potential was analyzed on an Agilent NovoCyte Quanteon using a 637-nm laser and emission was measured using a 695/40 filter.

### Mitochondrial volume analysis

HFFs seeded in 35 mm glass-bottom microscopy dishes were infected with ATPTG8Ty/cKD or ATPTG9Ty/cKD parasites expressing SOD2-GFP and IMC1-TdTomato. Parasites were treated with ethanol (vehicle control) for 72 h or 0.5 µg/mL ATc for 24, 48, or 72 h. Images of vacuoles containing either one or two parasites were acquired using the 60× lens of a DeltaVision II Microscope System II. Throughout imaging, parasites were maintained at 37°C. Each vacuole was imaged using a z-stack of 25 images spaced 0.2 µm apart such that the top and bottom images of the stack showed slightly out-of-focus mitochondria. Vacuoles containing parasites undergoing endodyogeny, as observed by the appearance of daughter cells using the IMC1-TdTomato signal, were excluded from analysis. Images were analyzed, and mitochondrial volume was calculated using the MitoGraph system as previously described (37). The MitoGraph software and ImageJ macros were downloaded according to directions available by the creators of MitoGraph (36). Each z-stack of images was opened in Fiji. The “GenFramesMaxProjs.ijm” macro was run to create a file in which the maximum intensity from each slice of the z-stack was projected. Using this image, a region of interest (ROI) was drawn around each vacuole of parasites in the field. The ROI was saved as an RoiSet.zip file and the “CropCells.ijm” macro was run to create individual stack files of each previously drawn ROI. Terminal was opened and navigated to the folder containing the previously downloaded MitoGraph software. The MitoGraph program was run via terminal by specifying the path to the folder containing the data as well as other critical parameters, such as the *x*/*y* pixel size (0.1069) and the spacing between z-stack images (0.2 µm). At least 100 vacuoles were analyzed over two or three biological replicates for each strain.

### Transmission electron microscopy

In order to quantify changes in mitochondrial cristae density upon CHCH domain subunit knockdown, HFF cells infected with parental + 72 h ATc, ATPTG8Ty/cKD ± 72 h ATc, or ATPTG9Ty/cKD ± 72 h ATc parasites were trypsinized, pelleted, and then fixed in a 50 mM phosphate (Sigma Aldrich cat. no. P5655-100G; Sigma Aldrich cat. no. P9666-100G) buffer containing 1% glutaraldehyde (Fisher Scientific. cat. no. NC1536477) and 1% OsO_4_ (Fisher Scientific, cat. no. NC9402523) for 45 min at 4°C. Samples were washed three times in cold 50 mM phosphate buffer and then rinsed in cold dH_2_O. Staining and imaging were conducted as previously described (12). En bloc staining was performed with 1% aqueous uranyl acetate (Ted Pella Inc.) at 4°C for 3 h. After rinsing with dH_2_O, a series of ethanol solutions was used to dehydrate the samples before they were embedded in Eponate 12 resin (Ted Pella Inc.). Ninety-five nanometers of sections was obtained using a Leica Ultracut UCT ultramicrotome (Leica Microsystems Inc.) before staining with lead citrate and uranyl acetate. Samples were viewed using a JEOL 1200 EX transmission electron microscope (JEOL USA Inc.), and images were captured using an AMT 8-megapixel digital camera and AMT Image Capture Engine V602 software (Advanced Microscopy Techniques). For cristae quantification, 60 sections of each strain that were pre-selected to contain parasite mitochondria were blinded. Fiji software was utilized to measure mitochondrial area, and cristae were counted manually. Images were subsequently un-blinded, and a student’s *t*-test was utilized to determine differences in cristae density and mitochondrial area between strains.

## SUPPLEMENTAL MATERIAL

Figure S1 mbio.01769-23-s0001.tif Promoter replacement results in modified ATPTG8 and ATPTG9 transcript levels.

Figure S2 mbio.01769-23-s0002.tif Immunoprecipitation of ATPTG8Ty and ATPTG9Ty.

Figure S3 mbio.01769-23-s0003.tif CHCH domain protein knockdown results in reduced mitochondrial membrane potential.

Figure S4 mbio.01769-23-s0004.tif Mitochondrial areas measured during transmission electron microscopy analysis.

Figure S5 mbio.01769-23-s0005.tif Stable exogenous expression of wildtype and cysteineΔserine ATPTG8.

Supplemental legends mbio.01769-23-s0006.docx Legends for Table S1 and Fig. S1 to S5.

Table S1 mbio.01769-23-s0007.xlsx Summary of oligonucleotides used in the present study.

ASM does not own the copyrights to Supplemental Material that may be linked to, or accessed through, an article. The authors have granted ASM a non-exclusive, world-wide license to publish the Supplemental Material files. Please contact the corresponding author directly for reuse.
