# Supplementary figures and images for: ATP synthase-associated coiled-coil-helix-coiled-coil-helix (CHCH) domain-containing proteins are critical for mitochondrial function in Toxoplasma gondii
Source: mBio. 2023 Oct 5;14(5):e01769-23. doi: 10.1128/mbio.01769-23 (PMC10653836; doi:10.1128/mbio.01769-23)

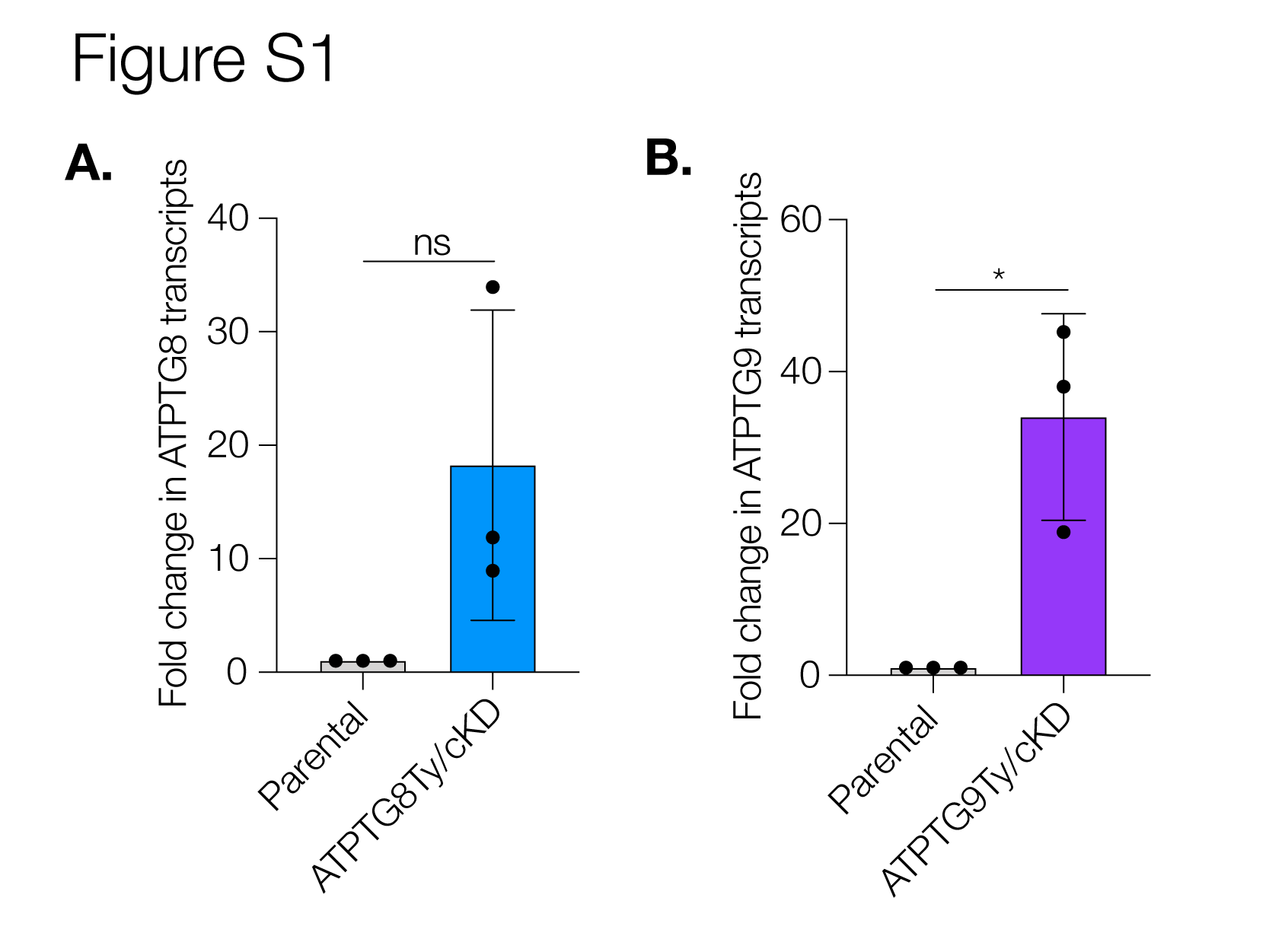

Supplement: Figure S1 — Promoter replacement results in modified ATPTG8 and ATPTG9 transcript levels. [file mbio.01769-23-s0001.tif]

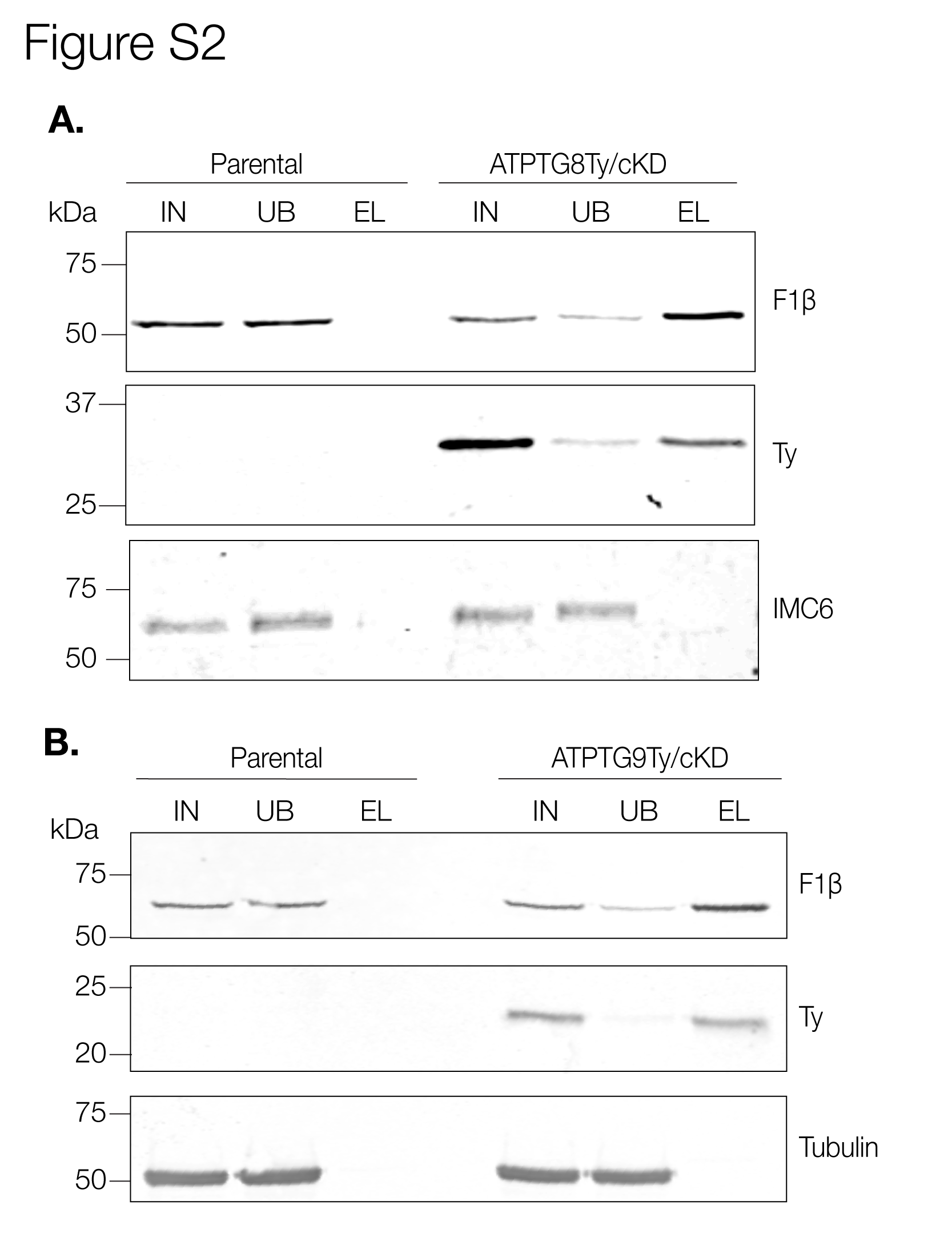

Supplement: Figure S2 — Immunoprecipitation of ATPTG8Ty and ATPTG9Ty. [file mbio.01769-23-s0002.tif]

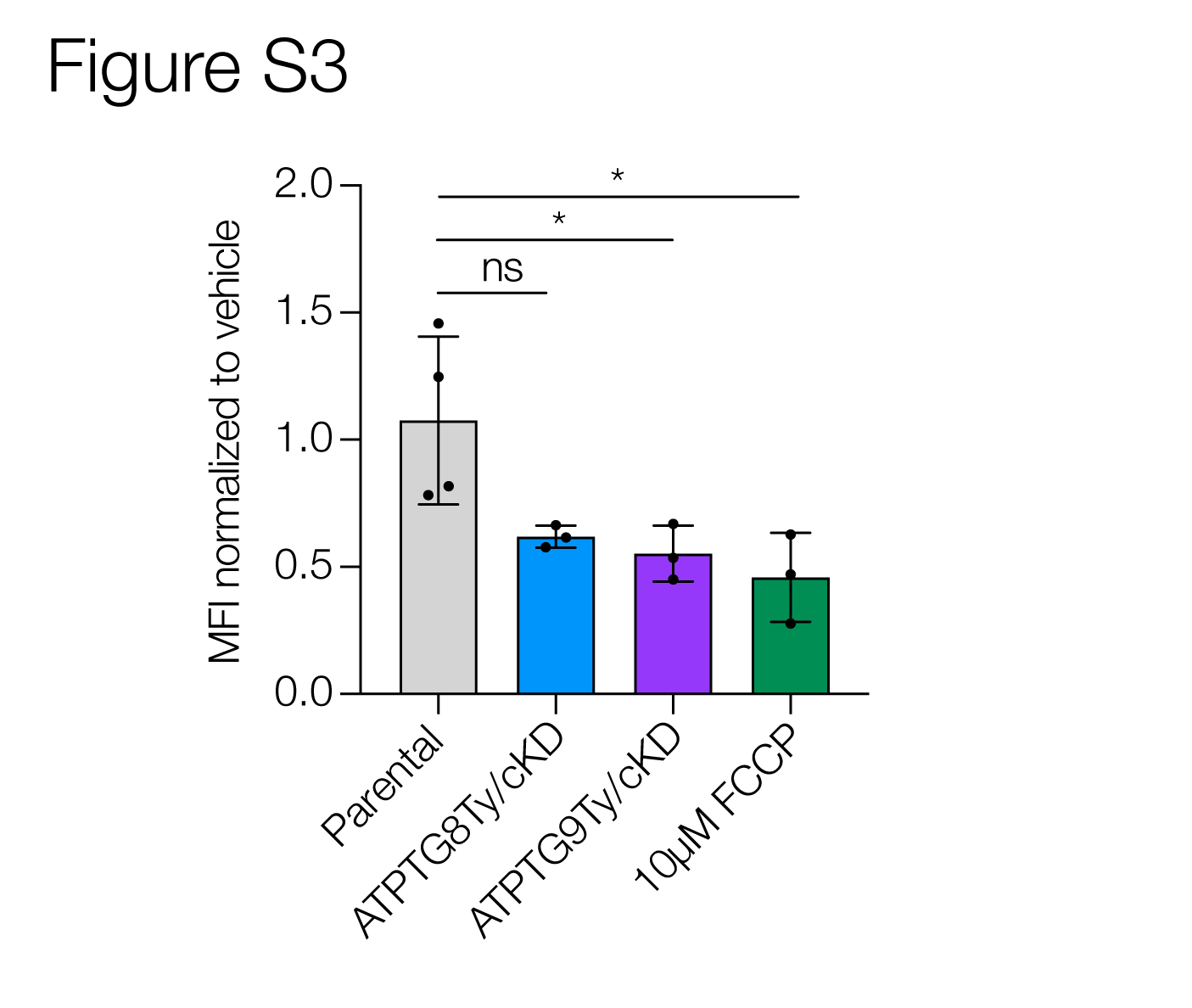

Supplement: Figure S3 — CHCH domain protein knockdown results in reduced mitochondrial membrane potential. [file mbio.01769-23-s0003.tif]

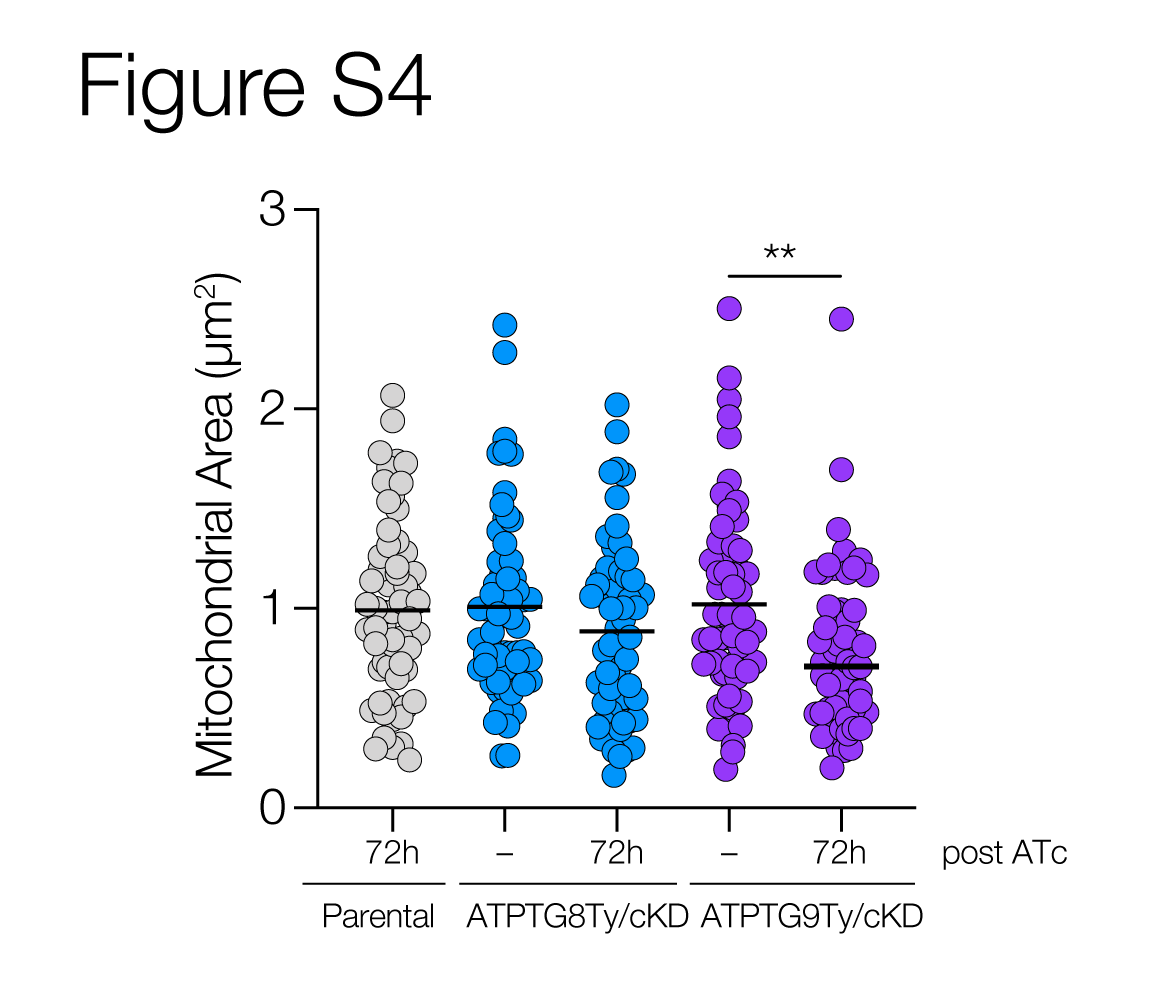

Supplement: Figure S4 — Mitochondrial areas measured during transmission electron microscopy analysis. [file mbio.01769-23-s0004.tif]

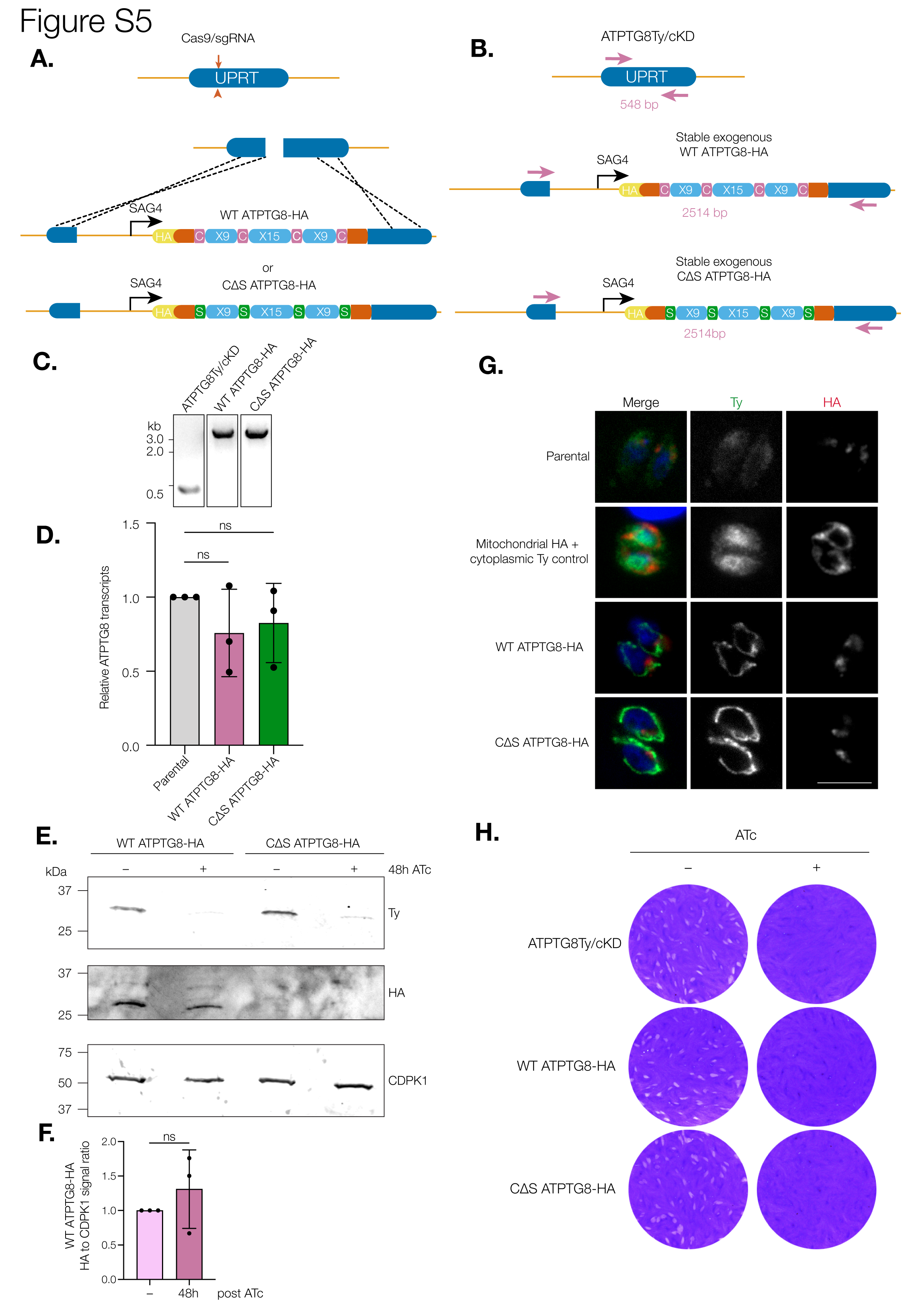

Supplement: Figure S5 — Stable exogenous expression of wildtype and cysteineΔserine ATPTG8. [file mbio.01769-23-s0005.tif]
