# Supplementary material for: ATP synthase-associated coiled-coil-helix-coiled-coil-helix (CHCH) domain-containing proteins are critical for mitochondrial function in Toxoplasma gondii
Source: mBio. 2023 Oct 5;14(5):e01769-23. doi: 10.1128/mbio.01769-23 (PMC10653836; doi:10.1128/mbio.01769-23)
Supplement: Supplemental legends — Legends for Table S1 and Fig. S1 to S5. [file mbio.01769-23-s0006.docx]

**Supporting information**

**Supplemental Table 1: Summary of oligonucleotides used in the present study.**

**Supplemental Figure 1: Promoter replacement results in modified ATPTG8 and ATPTG9 transcript levels. A.** Expression of ATPTG8 transcripts in ATPTG8Ty/cKD and parental parasites was analyzed via RT-qPCR using the primers P25 and P26. Actin was used as a housekeeping gene (P23 and P24). Three technical replicates were used over three biological replicates for each strain. Expression levels were normalized to the parental strain using the 2^–∆∆Ct^ method. Un-paired, two-tailed t-test (ns = not significant). **B.** Expression of ATPTG9 transcripts in ATPTG9Ty/cKD and parental parasites was analyzed via RT-qPCR using the primers P27 and P28. Actin was used as a housekeeping gene (P23 and P24). Three technical replicates were used over three biological replicates for each strain. Expression levels were normalized to the parental strain using the 2^–∆∆Ct^ method. Un-paired, two-tailed t-test (ns = not significant, p = 0.01 to 0.05: *).

**Supplemental Figure 2: Immunoprecipitation of ATPTG8Ty and ATPTG9Ty.** Following anti-Ty immunoprecipitation, the input (IN), unbound (UB), and elution (EL) fractions from parental and ATPTG8Ty/cKD **(A)** or ATPTG9Ty/cKD **(B)** parasites were analyzed by western blot. Membranes were probed with antibodies against Ty, F1β, and IMC6 **(A)** or tubulin **(B)**. Representative of at least three biological replicates.

**Supplemental Figure 3: CHCH domain protein knockdown results in reduced mitochondrial membrane potential.** Membrane potential of parental, ATPTG8Ty/cKD, and ATPTG9Ty/cKD parasites treated with ATc or vehicle control for 72h, or parental parasites treated with 10µM FCCP or vehicle control (DMSO) for 1 hour. Parasites were stained with 50nM MitoTracker and membrane potential was measured via flow cytometry. Mean fluorescence intensity (MFI) for each treatment condition was normalized to its vehicle control. Results represent mean ± SD for 3 independent replicates of ATPTG8Ty/cKD, ATPTG9Ty/cKD, and FCCP treatments or 4 independent replicates of parental strains. Unpaired, two-tailed t-test (ns = not significant, p = 0.01 to 0.05: *).

**Supplemental Figure 4: Mitochondrial areas measured during transmission electron microscopy analysis.** Quantification of mitochondrial area (µm^2^) from parental parasites +72h ATc and ATPTG8Ty/cKD or ATPTG9Ty/cKD treated with ATc or vehicle control (-) for 72h as part of Figure 4. Data represent mean ± SD for 60 sections of each condition, which were blinded prior to analysis in Fiji. Unpaired, two-tailed t-test (p = 0.001 to 0.01: **).

**Supplemental Figure 5: Stable exogenous expression of wildtype and cysteineΔserine ATPTG8**. **A.** Schematic of the strategy to stably express either a wildtype copy of ATPTG8 (WT), or a copy in which all cysteine residues were mutated to serines (CΔS). The two versions were targeted to the UPRT locus (TGGT1_312480). Parasites were transfected with an sgRNA targeting the aforementioned locus and a repair template containing a SAG4 promoter, an in-frame N-terminal HA tag, the ATPTG8 wildtype or CΔS CDS, followed by 1000bp of the ATPTG8 endogenous 3’ UTR and flanked with 40bp homology arms to the UPRT locus. **B.** Schematic of primers used to screen for positive integrants via PCR (P19 and P20). **C.** Following selection with 5-fluoro-2'-deoxyuridine (FUDR) and subcloning via serial dilution, integration of the repair template into the correct loci of WT ATPTG8-HA and CΔS ATPTG-HA parasite clones was confirmed with the primers shown in Figure S3B (P19 and P20). **D.** Expression of HA-tagged ATPTG8 transcripts in WT ATPTG8-HA and CΔS ATPTG-HA parasites was compared to the expression of Ty-tagged ATPTG8 transcripts in the parental line (ATPTG8Ty/cKD) via RT-qPCR. HA-tagged ATPTG8 transcripts were amplified using the primers P21 and P22 and Ty-tagged ATPTG8 transcripts were amplified using P29 and P22. Actin was used as a housekeeping gene (P23 and P24). Three technical replicates were used over three biological replicates for each strain. Expression levels of HA-ATPTG8 in WT ATPTG8-HA and CΔS ATPTG-HA parasites were normalized to Ty-ATPTG8 levels in the parental ATPTG8Ty/cKD strain using the 2^–∆∆Ct^ method. Un-paired, two-tailed t-test (ns = not significant). **E.** Lysates from ATPTG8Ty/cKD, WT ATPTG8-HA, and CΔS ATPTG8-HA parasites were prepared following treatment with ATc or vehicle control for 48h. Samples were separated via SDS-PAGE then probed with antibodies against HA, Ty, and CDPK1. Data are representative of three biological replicates. **F.** Densitometric analysis of HA signal in WT ATPTG8-HA parasites treated with ATc for 48h as normalized to parasites treated with vehicle control (-). CDPK1 levels used as a loading control. Unpaired, two-tailed t-test (ns = not significant). **G.** Intracellular ATPTG8Ty/cKD, WT ATPTG8-HA, and CΔS ATPTG8-HA parasites were stained for Ty (green), HA (red) and DNA (blue). Scale bar: 5µm. Data are representative of three biological replicates. **H.** ATPTG8Ty/cKD, WT ATPTG8-HA, and CΔS ATPTG8-HA parasites were allowed to grow undisturbed on an HFF monolayer for 7-8 days with either ATc or vehicle control (-). Data are representative of three biological replicates.
